# SIAH2 regulates DNA end resection and replication fork recovery by promoting CtIP ubiquitination

**DOI:** 10.1093/nar/gkac808

**Published:** 2022-09-26

**Authors:** Seo-Yeon Jeong, Gurusamy Hariharasudhan, Min-Ji Kim, Ji-Yeon Lim, Sung Mi Jung, Eun-Ji Choi, In-Youb Chang, Younghoon Kee, Ho Jin You, Jung-Hee Lee

**Affiliations:** Laboratory of Genomic Instability and Cancer therapeutics, Chosun University School of Medicine, 309 Pilmun-daero, Dong-gu, Gwangju 61452, Republic of Korea; Department of Cellular and Molecular Medicine, Chosun University School of Medicine, 309 Pilmun-daero, Dong-gu, Gwangju 61452, Republic of Korea; Laboratory of Genomic Instability and Cancer therapeutics, Chosun University School of Medicine, 309 Pilmun-daero, Dong-gu, Gwangju 61452, Republic of Korea; Laboratory of Genomic Instability and Cancer therapeutics, Chosun University School of Medicine, 309 Pilmun-daero, Dong-gu, Gwangju 61452, Republic of Korea; Laboratory of Genomic Instability and Cancer therapeutics, Chosun University School of Medicine, 309 Pilmun-daero, Dong-gu, Gwangju 61452, Republic of Korea; Department of Pharmacology, Chosun University School of Medicine, 309 Pilmun-daero, Dong-gu, Gwangju 61452, Republic of Korea; Laboratory of Genomic Instability and Cancer therapeutics, Chosun University School of Medicine, 309 Pilmun-daero, Dong-gu, Gwangju 61452, Republic of Korea; Department of Cellular and Molecular Medicine, Chosun University School of Medicine, 309 Pilmun-daero, Dong-gu, Gwangju 61452, Republic of Korea; Laboratory of Genomic Instability and Cancer therapeutics, Chosun University School of Medicine, 309 Pilmun-daero, Dong-gu, Gwangju 61452, Republic of Korea; Department of Cellular and Molecular Medicine, Chosun University School of Medicine, 309 Pilmun-daero, Dong-gu, Gwangju 61452, Republic of Korea; Department of Anatomy, Chosun University School of Medicine, 309 Pilmun-daero, Dong-gu, Gwangju 61452, Republic of Korea; Department of New Biology, Daegu Gyeongbuk Institute of Science and Technology (DGIST), 333 Techno-Joongang-daero, Dalseong-gun, Daegu 42988, Republic of Korea; Laboratory of Genomic Instability and Cancer therapeutics, Chosun University School of Medicine, 309 Pilmun-daero, Dong-gu, Gwangju 61452, Republic of Korea; Department of Pharmacology, Chosun University School of Medicine, 309 Pilmun-daero, Dong-gu, Gwangju 61452, Republic of Korea; Laboratory of Genomic Instability and Cancer therapeutics, Chosun University School of Medicine, 309 Pilmun-daero, Dong-gu, Gwangju 61452, Republic of Korea; Department of Cellular and Molecular Medicine, Chosun University School of Medicine, 309 Pilmun-daero, Dong-gu, Gwangju 61452, Republic of Korea

## Abstract

Human CtIP maintains genomic integrity primarily by promoting 5′ DNA end resection, an initial step of the homologous recombination (HR). A few mechanisms have been suggested as to how CtIP recruitment to damage sites is controlled, but it is likely that we do not yet have full understanding of the process. Here, we provide evidence that CtIP recruitment and functioning are controlled by the SIAH2 E3 ubiquitin ligase. We found that SIAH2 interacts and ubiquitinates CtIP at its N-terminal lysine residues. Mutating the key CtIP lysine residues impaired CtIP recruitment to DSBs and stalled replication forks, DSB end resection, overall HR repair capacity of cells, and recovery of stalled replication forks, suggesting that the SIAH2-induced ubiquitination is important for relocating CtIP to sites of damage. Depleting SIAH2 consistently phenocopied these results. Overall, our work suggests that SIAH2 is a new regulator of CtIP and HR repair, and emphasizes that SIAH2-mediated recruitment of the CtIP is an important step for CtIP’s function during HR repair.

## INTRODUCTION

DNA double-strand breaks (DSBs) are the most dangerous lesions that arise endogenously or by exposure to DNA damaging agents such as radiation, carcinogens and replication stress. If unrepaired or repaired incorrectly, DSBs can lead to genome instability, chromosome rearrangement, and cell death. Indeed, impaired DSBs repair is associated with developmental defects, immunological abnormalities, neurodegeneration, premature aging and cancer (1–3). To eliminate the risk of DSBs, the DNA damage response (DDR) system is activated to not only sense and repair DSBs, but also to coordinate cell cycle progression, transcription and cell death, based on cellular physiological status (4,5). In eukaryotic cells, nonhomologous end-joining (NHEJ) and homologous recombination (HR) are two key pathways responsible for the repair of DSBs. In the G1 phase, DSBs are quickly repaired by NHEJ, which is an error-prone solution since the two broken DNA ends are ligated without respect to sequence homology. On the other hand, HR uses a sister chromatid as a template when repairing broken DNA during the S and G2 phases, and thus is an error-free pathway for repairing DSBs and maintaining genome integrity (3,6,7). Repair of DSBs by HR is a multi-step pathway that is always initiated by DNA end resection, which involves a 5′ to 3′ nucleolytic degradation of one strand of the broken DNA end, generating short 3′-single stranded DNA (ssDNA) overhangs. The 3’ ssDNA overhangs are coated by replication protein A (RPA), which is subsequently displaced by RAD51, mediating homology search and invasion into the sister chromatid strand (8,9).

A key protein that is involved in the regulation of DNA end resection is C-terminal binding protein (CtBP) interacting protein (CtIP). CtIP is associated with the MRE11–RAD50–NBS1 (MRN) complex, which promotes the endonuclease activity of MRE11 to generate the short ssDNA overhangs (10–12). Following initial resection, EXO1 nuclease and DNA2 helicase, in complex with Bloom syndrome helicase (BLM), further extend the 3′-ssDNA. In particular, CtIP promotes DNA unwinding by BLM and the motor activity of DNA2 that accelerates the degradation of ssDNA (10,13–16). The presence of ssDNA is a potent inhibitor of NHEJ (8,17). Therefore, CtIP-derived end resection is a critical step for HR-mediated DNA repair and also a key determinant in the choice of pathways for DSB repair. In addition to its role in DNA end resection, CtIP has recently been shown to play a role at the replication fork during replication stress conditions (18–20). For example, CtIP is enriched at ongoing replication forks (21) and was shown to interact with proliferating cell nuclear antigen (PCNA), a DNA polymerase clamp involved in DNA replication that targets CtIP to regions of active DNA replication (22). CtIP has been identified as a replication fork protection factor because it prevents excessive DNA2-dependent nascent strand degradation in response to replication stress (18), and both the CtIP–BRCA1 complex and MRE11 play critical roles in maintaining replication fork progression (20).

The dynamic modulation of post-translation modifications (PTMs) tightly controls the activity of DNA repair proteins including CtIP (23–33). For instance, APC/CC^dh1^ and Cullin3-KLHL15 promote ubiquitination and degradation of CtIP, thereby facilitating the clearance of DNA damage-induced CtIP foci and inhibiting DNA end resection (27,28). RNF138 stimulates ionizing radiation (IR)-induced CtIP ubiquitination, which is important for accumulation at DSBs (33). USP52 de-ubiquitinates CtIP to promote DNA end resection and HR (32). The function of CtIP in end resection and HR is regulated by RNF138 and USP52 through ubiquitination. However, unlike other regulatory proteins, these do not affect the stability of CtIP. In addition, sumoylation of CtIP by CBX4 facilitates DNA end resection (23), whereas neddylation inhibits CtIP-mediated end resection (31). More recently, it has been reported that sumoylation of CtIP prevents over-resection of newly synthesized DNA at stalled replication forks (30). Therefore, the function of CtIP in end resection and replication fork stability is tightly controlled by several different PTMs.

Human SIAH proteins include two homologs, SIAH1 and SIAH2, which are evolutionarily conserved E3 ubiquitin ligases and have high sequence similarity in the N-terminal RING domain, two Zinc finger domains, and a C-terminal SBD domain, reflecting overlapping roles in the cell. However, since SIAH1 and SIAH2 differ in terms of substrate specificity and transcriptional regulation, it is thought that there are both distinct and overlapping biological functions (34). For example, although activated p53 induces expression of both SIAH1 and SIAH2 (35,36), only SIAH2 is induced by estrogen (37), hypoxia (38) and cytokine signaling (39). Moreover, ATM and ATR (40), mitogen-activated protein kinase MLK1 (41) and apoptosis-regulating kinase ASK1 (42) phosphorylate SIAH1, while dual specificity tyrosine phosphorylation regulated kinase 2 (DYRK2) (43), checkpoint kinase 2 (CHK2) (44) and p38 MAPK (45) phosphorylate SIAH2. ATM, a master regulator of DDR, phosphorylates SIAH1 at serine 19, disrupting interactions with homeodomain-interacting protein kinase 2 (HIPK2), a key regulator of the apoptotic program induced by DNA damage, and leading to p53-mediated apoptosis (40). However, the equivalent serine in SIAH2 (serine 16) is phosphorylated by DYRK2, which stimulates its ability to trigger the hypoxic response pathway, including angiogenesis (43).

In this study, we identified SIAH2 as a novel regulator of CtIP that controls the activity of CtIP in HR-mediated DSB repair and replication fork recovery through ubiquitination. Unlike SIAH1, which inhibits DSB repair (46), SIAH2 plays an important role in preventing the catastrophic DNA damage caused by DSB induction and replication stress by promoting DNA end resection and recovery of stalled replication forks. Our results point to a critical role for SIAH2 in promoting CtIP functioning to maintain chromosome stability.

## MATERIALS AND METHODS

### Cell culture and treatment

The human cervix adenocarcinoma cell line HeLa and human embryonic kidney HEK293T cells were cultured in Dulbecco's modified Eagle's medium (Invitrogen, USA) supplemented with 10% heat-inactivated fetal bovine serum (FBS; Invitrogen), 100 units/ml penicillin and 100 μg/ml streptomycin (Invitrogen). Cells were obtained from the American Type Culture Collection (ATCC, Manassas, VA, USA) and maintained in a humidified incubator containing 5% CO_2_ at 37°C. Cells in exponential growth were harvested for subsequent experiments. To induce DNA double strand breaks, exponentially growing cells were irradiated from ^137^Cs source at dose of 5 or 10 Gy (Gammacell 3000 Elan; Best Theratronic, Ottawa, Canada) and allowed to recover at 37°C incubator for various times. To replication stress, growing cells were treated with 3 mM hydroxyurea (HU; Sigma-Aldrich, USA) and then incubated at 37°C for indicated time points. For preventing protein degradation, cells were treated with proteasome inhibitors MG132 (Sigma-Aldrich) at 10 μm concentration for 4 h before harvesting.

### Plasmid construction and transfection

**Figure 1.**
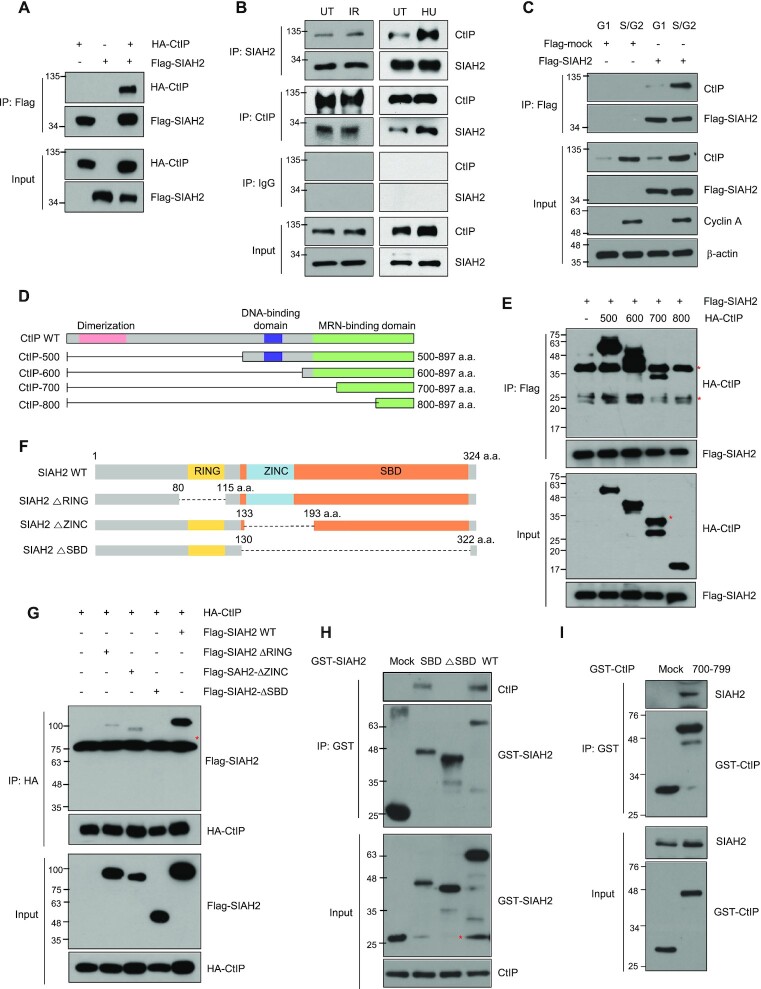
SIAH2 interacts with CtIP. (**A**) HEK293T cells were transfected with Flag-SIAH2 along with or without HA-CtIP. Total cell lysates were immunoprecipitated with either control IgG or anti-Flag antibody and then detected with the indicated antibodies. (**B**) HeLa cells were lysed either 30 min after ionizing radiation (IR) or 3 h after hydroxyurea (HU) treatment and subjected to immunoprecipitation followed by immunoblotting with the indicated antibodies. (**C**) Interaction between Flag-SIAH2 and endogenous CtIP in either G1 or S/G2 phase of the cell cycle. Hela cells transfected with Flag-mock or Flag-SIAH2 were synchronized in G1 and S/G2, cell lysates were immunoprecipitated with anti-Flag antibody and immunoblotting was performed with the indicated antibodies. (**D**) Schematic representation of the mutant CtIP constructs used in this study (**E**) HEK293T cells were cotransfected with each of the indicated HA-CtIP constructs and Flag-SIAH2 and subjected to immunoprecipitation and immunoblotting with the indicated antibodies. Asterisks indicate nonspecific bands. (**F**) Schematic representation of SIAH2 constructs used in this study. (**G**) Cellular lysates from HEK293T cells cotransfected with the indicated Flag-SIAH2 constructs and HA-CtIP, immunoprecipitated with anti-HA antibody and then immunoblotted with indicated antibodies. Asterisks indicate nonspecific bands. (**H**) Purified GST-SIAH2 WT, GST-SIAH2 ΔSBD, and GST-SIAH2 SBD expressed in bacteria were incubated with lysates from HeLa cells, and CtIP that coprecipitated with each GST-SIAH2 construct was analyzed by immunoblotting using the CtIP antibody. GST immunoblots show input GST-SIAH2 constructs. Asterisks indicate GST-SIAH2. (**I**) Purified GST-CtIP (700–799) expressed in bacteria was incubated with lysates from HeLa cells, and SIAH2 that coprecipitated with GST-CtIP (700–799) fragment was analyzed by immunoblotting using the SIAH2 and GST antibodies. GST immunoblots show input GST-CtIP constructs.

The full-length human wild-type CtIP was obtained by PCR from human HeLa cDNA and cloned into pcDNA3.1-Flag and pcDNA 3-HA vector. To prepare serial deletion constructs of CtIP-500, CtIP-600, CtIP-700 and CtIP-800, each fragment was PCR-amplified using pcDNA 3-HA-CtIP as template, and the PCR products were subcloned into a pcDNA 3-HA vector (Figure 1D; all amino acid sequences were based on the sequence of accession no. AAH30590.1). To generate CtIP 5KR mutant and 6KR mutant, pcDNA Flag-CtIP we performed mutagenesis using GENEART^®^ site-directed mutagenesis system (Thermo Fisher Scientific, USA) according to the manufacturer's instructions. The Myc-DDK-SIAH2 construct was obtained from OriGene (RC203802, OriGene Technologies, USA) and non-tagged SIAH2 was subcloned into a pcDNA3.1 vector. The SIAH2 WT, SIAH2 ΔRING, SIAH2 ΔZINC and SIAH2 ΔSBD constructs were amplified by PCR and cloned into the pFlag vector (Figure 1F; all amino acid positions were based on the sequence of accession NP_005058.3). Full length human SIAH1 cDNA was amplified HeLa cDNA, and the PCR products were cloned into pEGFP-N2 vector. For *in vitro* pull-down assay, the SIAH-SBD, SIAH2-ΔSBD and CtIP (aa 700–799) were cloned into the pEGX4T1 vector. The primer sequences used in these experiments were listed in Supplementary Table S1. All constructs were confirmed by automated DNA sequencing. Cells were transiently transfected with indicated constructs using lipofectamine 2000 (Invitrogen) following the manufacturer's instructions.

### siRNA sequences and Transfection

For knockdown of CtIP or SIAH2, HeLa cells were transiently transfected with siRNA using lipofectamine RNAiMax (Invitrogen). The sequences of siRNAs targeting CtIP and SIAH2 are as follows: CtIP siRNA, 5′-GCUAAAACAGGAACGAATCdTdT-3′, CtIP 3′UTR siRNA 5′-CAGAAGGATGAAGGACAGTTTdTdT-3′; SIAH2 siRNA #1, 5′-ACACAGCCATAGCACATCTTTdTdT-3′; SIAH2 siRNA #2 (3′-UTR), 5′-GCTGGCTAATAGACACTGAATdTdT-3′; RNF138 siRNA 5′-CCUGUGUCAAGAAUCAAAUdTdT-3′; BRCA1 siRNA 5′-CAGCUACCCUUCCAUCAUA-3′; SIAH1 siRNA, 5′-GAUAGGAACACGCAAGCAAdTdT-3′; Control siRNA sequences were 5′-CCUACGCCACCAAUUUCGUdTdT-3′.

### Western blotting and Immunoprecipitation analysis

Cells were lysed in RIPA buffer (50 mM Tris–HCl (pH 7.5), 150 mM NaCl, 1% NP-40, 0.5% sodium deoxycholate, 0.1% sodium dodecyl sulfate) with protease inhibitors (Roche Diagnostic Corp., USA). Equal amounts of protein were separated by 6–15% SDS-PAGE followed by electrotransfer onto a polyvinylidene difluoride membrane (Millipore). The membranes were blocked for 1 h with TBS-t (10 mM Tris–HCl (pH 7.4), 150 mM NaCl and 0.1% Tween-20) containing 5% skim milk and then incubated at 4°C with primary antibodies. The blots were washed four times for 15 min with TBS-t containing 0.1% Tween 20 and then incubated with peroxidase-conjugated secondary antibodies for 1 h. The membranes were then washed four more times and developed using an enhanced chemiluminescence detection system (ECL; GE Healthcare, USA). For cycloheximide chase analysis, HeLa cells were transfected with the SIAH2 siRNA or Flag-SIAH2 vector, and 48 h later, cells were treated with cycloheximide (Sigma, 10 g/ml) for the indicated times. The level of SIAH2 protein was quantified using ImageJ software. For the immunoprecipitation assays, cell extracts were precleared with Protein G-Sepharose beads (GE Healthcare) and then incubated with the appropriate antibodies at 4°C for 4 h. Next, fresh protein G-Sepharose beads were added and the reaction mixture was incubated overnight at 4°C with rotation. The beads were washed at least three times in RIPA buffer, resuspended in SDS sample buffer, and boiled for 5 min. Immune complexes were then analyzed by Western blotting. Western blot analysis we performed were repeated three times. The antibodies used in these experiments were listed in Supplementary Table S2.

### Immunofluorescence analysis

To visualize DNA damage foci, cells were grown on glass coverslips and were irradiated with 5 Gy of IR. The cells were then washed twice with PBS, fixed with 4% paraformaldehyde for 15 min and ice-cold 100% methanol for 5 min, followed by permeabilization with 0.3% Triton X-100 for 15 min at room temperature. Next, the cover slips were washed three times with PBS and then blocked with 5% BSA in PBS for 1 h. The cells were single or double immunostained with primary antibodies against various proteins overnight at 4°C. Next, the cells were washed with PBS and then stained with the Alexa Fluor 594, or/and Alexa Fluor 488 conjugated secondary antibodies (Molecular Probes, USA), as appropriate. After washing, the coverslips were mounted using Vectashield mounting medium with 4,6-diamidino-2-phenylindole (Vectashield, USA). For immunostaining of RPA, cells were pre-extracted for 2 min on ice with CSK buffer (10 mM PIPES, pH7.0, 100 mM NaCl, 300 mM sucrose, 3 mM MgCl_2_ and 0.5% Triton X-100), fixed with 4% paraformaldehyde for 15 min and permeablized with 0.4% Triton X-100 for 10 min at room temperature. The following steps were executed according to the standard immunostaining methods described above. Fluorescence images were taken using a confocal microscope (Zeiss LSM 900; Carl Zeiss, Germany) and analyzed with Zeiss ZEN Image software (Carl Zeiss).

### *In vitro* protein binding assay

For the *in vitro* binding assay of SIAH2 and CtIP, bacterially expressed GST-SIAH2 WT, GST-SIAH2 SBD, GST-SIAH2 ΔSBD, GST-CtIP (aa 700–799) or GST alone were immobilized onto Glutathion Sepharose 4B beads (GE Healthcare) and incubated with lysates prepared from HeLa cells for 3 h at 4°C. Cell lysates were prepared in TEN100 buffer (20 mM Tris–HCl (pH 7.4), 0.1 mM EDTA, 100 mM NaCl, 1 mM dithiothreitol, 1 mM phenylmethanesulfonyl fluoride, 10 μg/ml leupeptin and 10 μg/ml aprotinin). The GST beads were washed five times with NTEN buffer (0.5% NP-40, 20 mM Tris–HCl (pH 7.4), 1 mM EDTA and 300 mM NaCl), and bead-bound proteins were separated by SDS-PAGE and analyzed by western blotting using the CtIP, SIAH2 or GST antibodies.

### *In vivo* ubiquitination assay

After transfection with the indicated plasmids and/or siRNAs, cell lysates were prepared by lysing HeLa or HEK293T cells using ubiquitination buffer (1% NP40, 150 mM NaCl. 50 mM Tris–HCl (pH 8.0), 10 mM NaF, 1 mM Na_3_VO_4_, 5 mM EDTA, 1 mM EGTA, 1 mM DTT) with protease inhibitors, followed by pre-clearing with protein G Sepharose (GE Healthcare). The precleared lysates were incubated with anti-CtIP monoclonal antibody (61141, Active Motif) or anti-Flag polyclonal antibody (F7425, Sigma Aldrich) at 4°C overnight. Fresh G-Sepharose beads were added and incubated at room temperature for 4 h. The beads were washed three times with NP40 buffer (1% NP40, 50 mM Tris–HCl (pH 7.5), 150 mM NaCl, 5 mM EDTA) and buffer IV (200 mmol/l imidazole, 0.15 mol/l Tris–HCl (pH 6.7), 30% (v/v) glycerol, 0.72 mol/l β-mercaptoethanol, and 5% (w/v) SDS) was then added to the beads. The immunocomplexes were loaded on SDS-PAGE followed by western blotting with anti-HA monoclonal antibody (3715S, Cell Signaling) or anti-CtIP monoclonal antibody (9201S, Cell Signaling).

### Cell cycle analysis

The cell cycle distribution was determined by measuring the DNA content of individual cells by flow cytometry. Briefly, cells were harvested, washed with PBS, trypsinized and centrifuged at 1500 rpm for 5 min. The pellet was then resuspended and fixed in 1 ml of 70% cold ethanol. The fixed cells were washed with PBS, incubated with 10 mg/ml RNase A (Thermo Fisher Scientific Inc., Waltham, MA, USA) at 37°C for 30 min, stained with Propidium Iodide (PI, 50 mg/ml), and analyzed on a FACSCalibur flow cytometer (BD Biosciences, San Jose, CA, USA). The DNA content was measured determined using Cellquest Pro software (BD Bioscience).

### *In vivo* DNA end resection assay

Percentage of single strand DNA (ssDNA%) was measured in AID-DIVA (AID-AsiSI-ER) U2OS cells (gift from Dr Gaelle Legube) as described previously (47). Briefly, AsiSI-ER-U2OS cells were transfected with the indicated siRNAs and constructs. At 24 h after transfection, cells were treated with 700 nM 4-OHT (4-hydroxytamoxifen) for 4 h to induce AsiSI-dependent DSBs and genomic DNA was then extracted with a gDNA extraction kit (Bioneer). The 2–3 μg of genomic DNA was digested or mock digested with BsrGI or HindIII (New England Biolabs, UK) at 37°C overnight. This assay is based on the assumption that ssDNA generated by resection events surrounding the DSB site is resistant to digestion by restriction enzymes, which by definition require duplex DNA. The level of ssDNAs generated by DNA end resection at the specific AsiSI sites (DSBs) was evaluated by qPCR. The primer sequences used in these experiments were listed in Supplementary Table S1. △Ct was calculated by subtracting the Ct value of the mock-digested sample from the Ct value of the digested sample. The ssDNA% was calculated with the following equation: ssDNA% = 1/(2^(△Ct-1)^ + 0.5) × 100.

### Homologous recombination assay

HR was measured using DR-GFP U2OS cells as described previously (48). Briefly, DR-GFP U2OS cells were transfected with undicated siRNAs and/or constructs using Lipofectamine 2000 (Invtrogen). At 6 h after transfection, cells were transfected with the I-SceI expression vector using Turbofect transfection reagent (Thermo Fisher Scientific). After 2–3 days, the percentage of GFP-positive cells was determined by flow cytometry (BD FACSCalibur, USA). For each analysis, 10 000 cells were processed and each experiment was repeated three times.

### Chromosomal aberration analysis

Indicated cells were treated with 2 Gy of IR or 2 mM HU. At 24 h after treatment, cells were washed in PBS and treated with 100 ng/ml colcemid (Sigma-Aldrich) for 3 h at 37°C to arrest the cells in metaphase. Cells were then harvested by trypsinization, incubated in 75 mM KCl for 20 min at 37°C and fixed in a methanol/acetic acid (3:1) solution. After removal of supernatant, the pellets were resuspended in fixative solution, dropped onto slides to obtain chromosome spreads and air-dried overnight. The slide was mounted in with mounting medium with DAPI (Vectashield). The metaphase images were captured using confocal microscope (Zeiss LSM 900; Carl Zeiss) and visible chromatid breaks/gap were counted. At least 50 chromosomes were analyzed and representative images were shown.

### Clonal survival assay

After treatment with the indicated doses of IR or HU, 1 × 10^3^ cells were immediately seeded onto a 60 mm dish in triplicated and grown for 2 weeks at 37°C to allow colony formation. Colonies were fixed with 95% methanol for 10 min and stained with 2% methylene blue in 50% ethanol. The number of colonies were counted. The percentage of surviving cells was calculated as the ratio of the plating efficiencies of treated cells compared to untreated cells. Results of clonal survival were presented as the mean value ± standard deviation (SD) for three independent experiments.

### Chromatin fractionation

Control or SIAH2 siRNA-transfected HeLa cells were treated with IR (dose, time). The harvested cells were resuspended and incubated for 10 min in ice-cold buffer containing 10 mM HEPES–KOH (pH 7.9), 1.5 mM MgCl_2_, 10 mM KCl, 0.2% Triton X-100, and complete protease inhibitor cocktail (Roche Diagnostic Corp), and centrifuged at 400g for 15 min at 4°C. The supernatant containing the soluble fraction was collected and the pellet was washed in ice-cold PBS, resuspended in cold buffer containing 420 mM NaCl, 20 mM HEPES–KOH (pH 7.9), 20% glycerol, 2 mM MgCl_2_, 0.2 mM EDTA, 0.1% triton, 0.5 mM DTT and complete protease inhibitor cocktail (Roche Diagnostic Corp.), incubated on ice for 1 h. After centrifugation at 13 000 rpm for 30 min at 4°C, the chromatin-containing pellet was resuspended in cold PBS supplemented with 600 mM NaCl, 1% *N*-octyl glucoside, and 125 units of DNase, incubated for 30 min in an ultrasonic bath, and centrifuged for 15 min at 18 000g at 4°C. Chromatin proteins were collected with the supernatant.

### Chromatin immunoprecipitation (ChIP) assay

DR-GFP U2OS cells were transfected with indicated siRNAs and/or constructs for 48 h and then transfected with pCBA-I-SceI plasmid. After 8 h, ChIP was performed using a commercially available SimpleChIP Assay Kit (Cell Signaling Technology) according to the manufacturer's instructions. DNA-bound protein was immunoprecipitated using an anti-CtIP antibody (61141, Active motif), anti-Flag antibody (F7425, Sigma Aldrich), or rabbit IgG (Active Motif) as a negative control. For quantification of co-precipitated DNA, samples were amplified using primers. The primer used for the detection of the I-SceI break site were set hybridizing at a distance of 180 bp from DSB site. The enrichments were calculated using the percent input method that is the signals obtained from the ChIP samples are divided by the signals obtained from the input samples. Each reaction was performed in triplicate, and results of three independent experiments were used for statistical analysis. The primer sequences used in these experiments were listed in Supplementary Table S1.

### *In situ* interactions at replication forks (SIRF) assay

The SIRF assay was measured in cells, using methods previous described (49). HeLa cells were transfected with indicated siRNAs and/or constructs, treated with 125 μM EdU (Invitrogen) for 10 min, washed two times with PBS, and subsequently treated with 10 mM HU. After 3 h, cells were fixed, permeabilized with 0.25% Triton-X, and a click-iT reaction (Invitrogen) was performed using biotin azide (Life Technologies Corp.) according to manufacturer's instruction. Cells were then washed with PBS and blocked in blocking solution at 37ºC humidified chamber for 1 h. After washing with PBS, cells were incubated with two primary antibodies. The primary antibodies used were as followed: mouse monoclonal anti-Flag (Sigma-Aldrich, 1:100) and rabbit polyclonal anti-biotin (Cell Signaling, 1:100). The negative control was used no EdU treatment. After incubation with primary antibodies, anti-rabbit MINUS, and anti-mouse PLUS PLA probes (1:5 dilution, Duolink, Sigma-Aldrich) were used to detect two primary antibodies according to the manufacturer instruction. Briefly, coverslip was blocked in Duolink blocking buffer for 1 h at room temperature and incubated with two primary antibodies. Upon washing the coverslip twice in PBS for 5 min, anti-mouse PLUS and anti-rabbit MINUS PLA probes (Sigma-Aldrich) were coupled to the primary antibodies for 1 h at 37 ºC. Next, amplification using the ‘Duolink In Situ Detection Reagents Red’ (Sigma-Aldrich) was performed at 37°C. After amplification, coverslips were mounted using DAPI-containing mounting media (Vectashield) and imaged on a Carl Zeiss LSM 900 confocal microscope. Data are representative of three independent experiments.

### DNA fiber assay

For measurement of replication stalled fork, indicated HeLa cells were labeled with 50 μM IdU (Sigma-Aldrich) for 1 h, followed by exposure to 3 mM HU for 3 h, and then chased with 500 μM CIdU (Sigma-Aldrich) for 1 h. For measurement of fork degradation, cells were first labeled with 50 μM IdU for 30 min, followed by 500 μM CIdU for 30 min, and incubated with 3 mM HU for 2 h. After the labeled cells were harvested, cells were lysed in lysis buffer (50 mM EDTA, 0.5% SDS, 200 mM Tris–HCl pH 7.5) and DNA fibers were stretched onto glass slides and fixed in methanol: acetic acid (3:1). The coverslips were then denatured (2.5 M HCl for 45 min), neutralized (0.1 M sodium borate), and blocked (5% BSA and 0.5% Tween 20 in PBS for 30 min). The labeled IdU and CIdU tracks were revealed with primary antibodies recognizing IdU and CIdU, respectively. Anti-rat IgG conjugated with Alexa Fluor 488 for IdU and Alexa Fluor 594-conjugated anti-mouse IgG for CldU were used for secondary antibodies. Images were obtained using a confocal microscope (Zeiss LSM 900) and fiber lengths analyzed using Zeiss microscopic imaging software ZEN (Carl Zeiss).

### Statistical analysis.

All data were analyzed with GraphPad Prism software and Microsoft Excel. Differences between two independent groups were tested with two-tailed paired Student′s *t*-test. For the nonparametric statistical test, Mann–Whitney test was used. *P* value of <0.01 was considered statistically significant and *P* values were indicated by asterisks as followed: **P < 0.01 and n.s. = nonsignificant. Error bars represent standard deviation (SD) of three independent experiments. All experiments were performed in triplicate, and repeated at least three times.

## RESULTS

### SIAH2 interacts with CtIP

To better characterize the regulation of DNA end resection in human cells, we searched for proteins that interact with the C-terminal region of CtIP (aa 500–897). This region of CtIP associates with the MRN complex, a major regulator of DNA end resection, and modifications (phosphorylation and ubiquitination) in this region are known to influence DNA end resection (6,26,28,33). Using a yeast two-hybrid screen, we identified SIAH1 and SIAH2 as binding partners of CtIP. SIAH1 and SIAH2, RING E3 ubiquitin ligases (40,50), have been identified previously as proteins that interact with CtIP in a yeast two-hybrid screen (51). SIAH1 promotes CtIP degradation through the ubiquitin-proteasome pathway (51), and the DDR master regulators ATM and ATR phosphorylate SIAH1 to facilitate degradation of HIPK2, an important regulator of cell death (40). On the other hand, there are no reports of SIAH2 being involved in the DDR or regulating CtIP through ubiquitination, thus we explored the nature of the interaction further. The first step was to investigate the interactions between SIAH2 and CtIP. When wild-type (WT) FLAG-tagged SIAH2 and HA-tagged CtIP were expressed ectopically in HEK293T cells and subjected to coimmunoprecipitation, interactions between the two were detected (Figure 1A). Moreover, endogenous SIAH2 coimmunoprecipitated with endogenous CtIP in the presence or absence of treatment with IR and hydroxyurea (HU), and vice versa (Figure 1B). In normal cells, CtIP foci are typically observed in the S or G2 phases of the cell cycle unless the cells are exposed to DNA damaging agents. We prepared cell lysates from HeLa cells synchronized in either G1 or S/G2 phases and observed that the interaction can be detected specifically in S/G2 phase, as expected (Figure 1C).

To define the particular domain of CtIP that mediates the interaction with SIAH2, we generated a truncated construct including the C-terminal region of CtIP alone (aa 500–897; HA-CtIP-500) and three additional versions of this construct lacking the DNA-binding domain (HA-CtIP-600) or both the DNA-binding domain and the MRN-binding (HA-CtIP-700 and HA-CtIP-800) domains (Figure 1D). When WT and the three truncated mutants of HA-CtIP were compared, we concluded that SIAH2 interacts with the region including amino acids 700–799, which is located in the MRN-binding domain (Figure 1E). Similarly, we generated three Flag-SIAH2 truncation mutants lacking either the RING (Flag-SIAH2 ΔRING), ZINC (Flag-SIAH2 ΔZINC) or SBD (Flag-SIAH2 ΔSBD) domains (Figure 1F). Coimmunoprecipitation of HA-CtIP with either of the RING or ZINC Flag-SIAH2 truncation mutants was detectable but interactions were almost completely abrogated when SIAH2 was lacking the SBD domain (Figure 1G), indicating the importance of this substrate-binding domain for interactions with CtIP.

To confirm the interaction between SIAH2 and CtIP *in vitro*, we purified GST-SIAH2 WT, GST-SIAH2 SBD and GST-SIAH2 ΔSBD from bacterial cells. A pull-down assay using total cell extracts of HeLa cells revealed that GST-SIAH2 WT and GST-SIAH2 SBD interacted with CtIP, while GST-SIAH2 ΔSBD did not (Figure 1H). Noticeably, purified GST-CtIP (aa 700–799) readily interacted with SIAH2 (Figure 1I), pointing to the importance of this region of CtIP.

### SIAH2-mediated ubiquitination of CtIP facilitates recruitment to DSB sites

**Figure 2.**
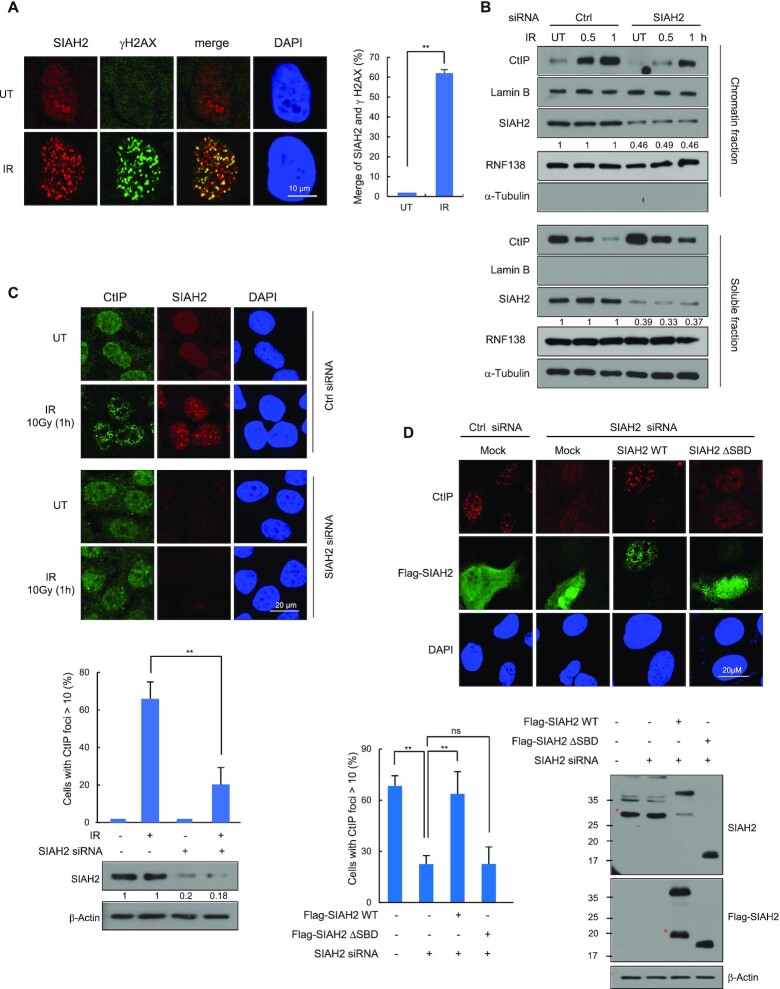
SIAH2 stimulates CtIP recruitment to DSB sites. (**A**) HeLa cells were either untreated or treated with 5 Gy of IR, fixed at 1 h, and immunostained with anti-SIAH2 and anti-γ-H2AX antibodies. Colocalization of SIAH2 (red) and γ-H2AX (green) in cells appears yellow in the merged image. DNA was stained with DAPI. The percent colocalization between SIAH2 and γ-H2AX is shown. Results are shown as the mean ± SD (*n* = 3), **P < 0.01, two-tailed Student's *t*-test. (**B**) Control and SIAH2-depleted HeLa cells were either untreated or treated with 5 Gy IR. At the indicated time point after IR, chromatin and soluble fractions were extracted and analyzed by western blotting. (**C**) Control and SIAH2-depleted HeLa cells were either untreated or treated with 5 Gy IR, fixed at 1 h, and immunostained with anti-CtIP and anti-SIAH2 antibodies. DNA was stained with DAPI. Representative images and the percentage of cells containing >10 CtIP foci are shown. The numbers were relative levels of SIAH2 expression compared to untreated control siRNA-transfected cells. Data represent mean ± SD (*n* = 3), **P < 0.01, two-tailed Student's *t*-test. (**D**) The number of CtIP foci was measured 1 h after IR treatment in control Hela cells or SIAH2-depleted HeLa cells reconstituted with Flag-Mock, Flag-SIAH2 WT, or Flag-SIAH2 ΔSBD. Representative images and the percentage of cells containing >10 CtIP foci in Flag-positive cells are shown. Asterisks indicate nonspecific bands.

Next, we wondered whether SIAH2 itself localizes to DSB sites. We used immunofluorescence staining to detect SIAH2 in cells that had been exposed to IR and observed discrete nuclear foci that co-localized with γ-H2AX (Figure 2A). When SIAH2 was depleted, the IR-induced SIAH2 foci were largely absent (Supplementary Figure S1A), indicating that the foci truly represent the SIAH2 proteins. SIAH2 is also found in the chromatin-enriched fractions, although the enrichment did not increase after IR unlike CtIP (Figure 2B). However, CtIP enrichment in the chromatin fraction was significantly reduced upon SIAH2 knockdown, consistently suggesting that SIAH2 is required for CtIP chromatin localization. (Figure 2B). Similarly, the IR-induced CtIP foci is significantly reduced upon SIAH2 knockdown in the S/G2-phase (Figure 2C and Supplementary Figure S1B). This effect is unlikely to be due to cell cycle changes, as SIAH2 knockdown did not majorly affect cell cycle distribution (Supplementary Figure S1C). The reduced foci formation of CtIP upon SIAH2 knockdown was nearly rescued by re-expressing FLAG-SIAH2 WT, but not by FLAG-SIAH2 ΔSBD, ruling out siRNA off-target effects and suggesting that the requirement of the SBD domain in the regulation (Figure 2D).

To test for similar effects on endonuclease-induced DSBs in SIAH2-depleted cells, we used the DR-GFP U2OS reporter system, in which a single DSB can be created at a specific site upon endonuclease I-SceI overexpression (48). ChIP-qPCR assays following I-SceI break site induction showed that CtIP recruitment to the DSB site was abrogated in SIAH2-depleted cells. Consistently, reconstitution of SIAH2 knockdown cells with Flag-SIAH2 WT, but not Flag-SIAH2 ΔSBD, restored CtIP recruitment to normal levels (Supplementary Figure S1D).

### SIAH2 induces CtIP ubiquitination

**Figure 3.**
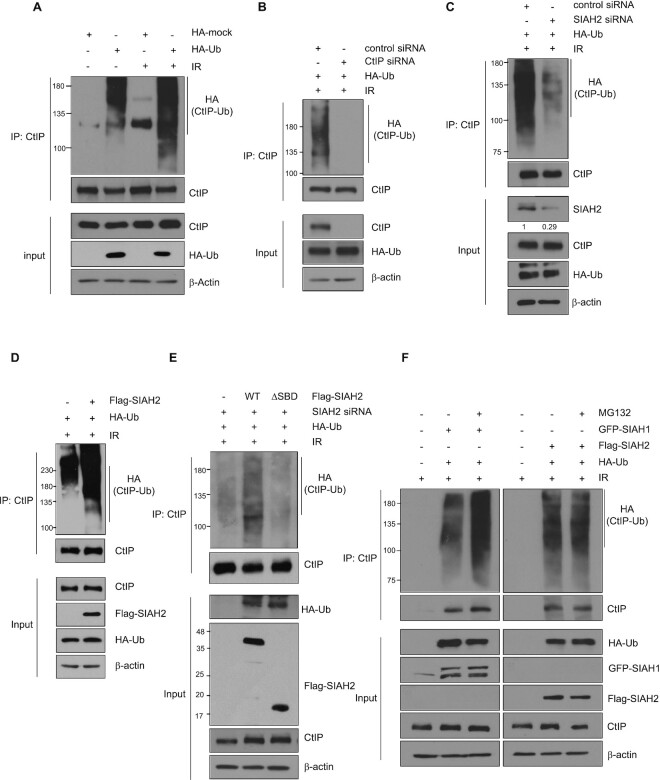
SIAH2 mediates CtIP ubiquitination. (**A**) HEK293T cells transfected with or without HA-ubiquitin were treated with or wihout 10 Gy IR for 30 min. Total cell lysates were immunoprecipitated with anti-CtIP antibody and immunoblotted with the indicated antibodies. (**B**) HEK293T cells co-transfected with HA-ubiquitin and either a control siRNA or CtIP siRNA were treated with 10 Gy IR for 30 min. Total cell lysates were immunoprecipitated with anti-CtIP antibody and immunoblotted with the indicated antibodies. (**C**) HEK293T cells co-transfected with HA-ubiquitin and either a control siRNA or SIAH2 siRNA were treated with 10 Gy IR for 30 minutes. Total cell lysates were immunoprecipitated with anti-CtIP antibody and immunoblotted with the indicated antibodies. The numbers were relative levels of SIAH2 expression compared to control siRNA-transfected cells. (**D**) HEK293T cells co-transfected with either Flag-Mock or Flag-SIAH2 along with HA-ubiquitin were treated with 10 Gy IR for 30 min. Total cell lysates were immunoprecipitated with anti-CtIP antibody and immunoblotted with the indicated antibodies. (**E**) SIAH2-depleted HEK293T cells co-transfected with Flag-Mock, Flag-SIAH2 WT, or Flag-SIAH2 ΔSBD along with HA-ubiquitin were treated with or without 10 Gy of IR for 30 min. Total cell lysates were immunoprecipitated with anti-CtIP antibody and immunoblotted with the indicated antibodies. Asterisks indicate nonspecific bands. (**F**) HEK293T cells co-transfected with HA-ubiquitin along with either GFP-SIAH1 or Flag-SIAH2 were treated with 10 Gy IR for 30 min. Cells were treated with or without 10 μm MG-132 for 4 h and total cell lysates were immunoprecipitated with anti-CtIP antibody and immunoblotted with the indicated antibodies.

Based on the evidence for direct interactions between SIAH2 and CtIP and the known ubiquitin E3 ligase function of SIAH2, we hypothesized that CtIP may be a substrate for SIAH2. First, we tested whether IR treatment would stimulate CtIP ubiquitination. First, we were able to detect high M.W. species of endogenous CtIP proteins when they are immunoprecipitated, in HA-ubiquitin transfected cells (Figure 3A lanes 1 and 2; detection of the high M.W. species was weaker if HA-ubiquitin is not expressed), as reported (33). The high M.W. species (ubiquitinated CtIP proteins) was increased when cells were treated with IR (compare lanes 2 and 4). Similar pattern is observed with exogenously expressed FLAG-CtIP proteins (Supplementary Figure S2A). The ubiquitination species nearly disappeared upon knockdown of CtIP, suggesting that they indeed represent ubiquitinated CtIP proteins (Figure 3B). Notably, when the cells were transfected with siRNA targeting SIAH2, IR-induced CtIP ubiquitination was significant decreased (Figure 3C). Likewise, when CtIP was immunoprecipitated from either control or SIAH2-overexpressing cells, a significantly higher level of ubiquitinated CtIP was isolated from the SIAH2-expressing cells upon IR exposure than from control cells (Figure 3D).

To further assess the impact of SIAH2 on CtIP ubiquitination, we knocked down SIAH2 using siRNA targeting the 3′ UTR region and reconstituted with ectopically expressed FLAG-SIAH2 WT or the FLAG-SIAH2 ΔSBD mutant in combination with HA-ubiquitin and then treated with IR. As shown in Figure 3E, complementation of SIAH2-depleted cells with Flag-SIAH2 WT restored CtIP ubiquitination. In contrast, little ubiquitination was detectable when Flag-SIAH2 ΔSBD was expressed, further supporting the importance of the SBD domain. Because ubiquitination and proteolysis are often coupled, we tested the effects of SIAH2 on CtIP stability and found that, although it has been reported that overexpression of Flag-SIAH1 reduces CtIP protein levels (51), there was little change in CtIP protein levels in SIAH2-overexpressing cells (Supplementary Figure S2B). Likewise, knockdown of SIAH2 using two different siRNA targeting SIAH2 (siSIAH2 #1 and #2) did not affect the expression levels of endogenous CtIP protein (Supplementary Figure S2C). A protein turnover study revealed that the half-life of CtIP was not altered when SIAH2 was depleted (Supplementary Figure S2D) or Flag-SIAH2 was overexpressed (Supplementary Figure S2E). We then looked at the efficiency of CtIP ubiquitination by SIAH1 and SIAH2 in the presence of MG-132, a proteasome inhibitor, to identify any role for proteosome-mediated degradation. SIAH1-mediated ubiquitination of CtIP increased in response to treatment with MG-132, whereas there was no effect on SIAH2-induced CtIP ubiquitination (Figure 3F), suggesting that SIAH2-mediated CtIP ubiquitination does not lead to degradation. It was reported that the ubiquitin E3 ligase RNF138, BRCA1, and SIAH1 promotes CtIP ubiquitination (29,33,51). Indeed, knocking down RNF138 also reduced the CtIP ubiquitination, to the level comparable to the SIAH2 knockdown (Supplementary Figure S2F; although RNF138 knockdown had lesser effect than that of SIAH2, it is hard to compare due the differences in the knockdown efficiency). Knockdown of SIAH1 or BRCA1 has lesser effect than that of SIAH2 knockdown (Supplementary Figure S2G). We conclude that similar to RNF138, SIAH2 is a major contributor to the CtIP ubiquitination (see Discussion).

### SIAH2 facilitates DNA end resection and HR

**Figure 4.**
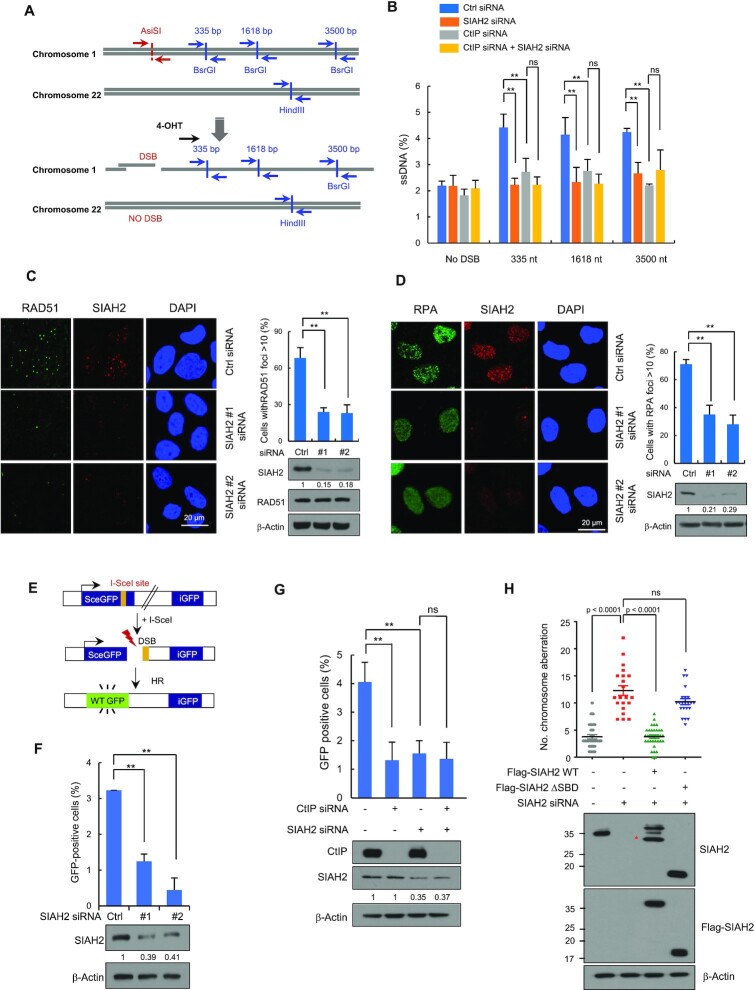
SIAH2 promotes DNA end resection and HR repair. (**A**) Schematic illustration of qPCR primers (blue arrow) used for measuring resection at sites adjacent to the AsiSI sites (red arrow). (**B**) Quantification of ssDNA generated by 5’ end resection at three AsiSI-induced DSBs in AsiSI-ER-U2OS cells transfected with either control siRNA or siRNAs against CtIP, SIAH2 or both SIAH2 and CtIP. Data represent mean ± SD (*n* = 3), **P < 0.01. ns, nonsignificant, two-tailed Student's *t*-test. (C and D) Control and SIAH2-depleted HeLa cells were treated with 5 Gy IR, fixed at 3 h, and immunostained with anti-RAD51 and anti-SIAH2 antibodies (**C**) or with anti-RPA and anti-SIAH2 antibodies (**D**). DNA was stained with DAPI. The percentage of cells containing >10 foci was calculated. Representative images and quantification of RAD51 and RPA foci are shown. Whole cell lysates were analyzed by immunoblotting using the indicated antibodies. Data represent mean ± SD (*n* = 3), **P < 0.01, two-tailed Student's *t*-test. (**E**) A schematic of the DR-GFP reporter system used to measure rates of homologous recombination (HR). (F and G) HR efficiency in control- and SIAH2-depleted DR-GFP-U2OS cells (**F**) and DR-GFP-U2OS cells transfected with the indicated siRNA combinations (**G**). The cells were transfected with an I-SceI expression plasmid for 48 h and HR efficiency was determined by FACS. An immunoblot confirming the presence of proteins after transfection is presented in the bottom panel. The numbers were relative levels of SIAH2 expression compared to control siRNA-transfected cells. Data represent mean ± SD (*n* = 3), **P < 0.01. ns, nonsignificant, two-tailed Student's *t*-test. (**H**) Quantification of chromosomal aberrations in either control or SIAH2-depleted HeLa cells reconstituted with Flag-Mock, Flag-SIAH2 WT or Flag-SIAH2 ΔSBD 24 h after treatment with 2 Gy of IR. Dot plot shows the number of chromosomal aberrations per cells. Data represent mean ± SD (*n* = 3) ns, nonsignificant. *P* values between indicated samples were calculated using a Mann–Whitney test. Asterisk indicates nonspecific band.

A major function of CtIP is resecting DNA ends at DSBs to generate ssDNA overhangs as the first step in HR (10). Because our results revealed that SIAH2 interacts with CtIP and promotes CtIP ubiquitination, we predicted that SIAH2 would influence the CtIP-mediated DNA end resection. To test this hypothesis, we measured the ssDNA generation at sites of DSBs induced by the AsiSI restriction enzyme using a previously developed AsiSI-ER-U2OS reporter system (47). Briefly, incubation of cells with 4-hydroxytamoxifen (4-OHT) facilitates the entry of AsiSI enzyme into the nucleus to induce DSBs, which are then repaired through DNA end-resection and HR. The percentage of resected DSBs was calculated by qPCR using three sets of primers that amplify three regions that include HindIII sites located 335, 1618 and 3500 bp from the AsiSI break sites (Figure 4A). Depletion of SIAH2 resulted in a significant decrease in DNA end-resection (Figure 4B and Supplementary Figure S3A). The efficiency of DNA end-resection at DSBs was similar in either SIAH2 or CtIP knockdown cells and knockdown of CtIP and SIAH2 together did not decrease DNA end resection efficiency any further, supporting the conclusion that SIAH2 functions in this process through CtIP.

During the process of resection, RPA coats ssDNA overhangs and is then replaced by RAD51 to facilitate HR-based DNA repair (9). We predicted that the process of HR repair would be impaired if DNA end resection were disrupted. Knockdown of SIAH2 significantly decreased the number of RAD51 (Figure 4C and Supplementary Figure S3B) and RPA foci (Figure 4D and Supplementary Figure S3C) in the S/G2-phase. We then examined the effect of SIAH2 on HR repair. HR efficiency was measured in a SIAH2 knockdown in U2OS cells carrying the integrated DR-GFP reporter assay (48). In this system, DSBs are generated through the expression of I-SceI endonuclease, which cleaves a specific recognition site in the gene encoding GFP, and the efficiency of HR repair is measured as the percentage of cells expressing GFP as detected by flow cytometry (Figure 4E). The levels of HR were measured in DR-GFP-U2OS cells depleted in SIAH2 and the percentage of GFP-positive cells was ∼3-fold (p < 0.01) lower than in control cells (Figure 4F) and similar to that of a CtIP knockdown (Figure 4G). This effect was not due to reduced S/G1 phase as SIAH2 knockdown did not affect cell cycle profiling in DR-GFP-U2OS cells (Supplementary Figure S3D). Notably, knockdown of both CtIP and SIAH2 showed a low HR efficiency similar to that of single knockdown (Figure 4G, last lane), again indicating that SIAH2 and CtIP function in the same HR repair pathway.

To further explore a role for SIAH2 specifically in the repair of DSBs, we exposed cells to IR and then measured levels of γ-H2AX, a marker of DSB, using immunofluorescence. A SIAH2 knockdown clearly showed higher numbers of unrepaired DSBs after exposure to IR, as evidenced by the increased number of γ-H2AX foci compared to the control (Supplementary Figure S3E). Moreover, clonogenic survival assays performed on both control and SIAH2-depleted cells showed that SIAH2 depletion rendered cells more sensitive to IR (Supplementary Figure S3F). These data suggest that SIAH2 depletion results in impaired DNA end resection and an HR repair phenotype.

If SIAH2 plays a role in the HR repair pathway then it may also influence chromosome stability. To test this, control HeLa cells, SIAH2-depleted HeLa cells, and SIAH2-depleted HeLa cells reconstituted with either FLAG-SIAH2 WT or FLAG-SIAH2 ΔSBD were exposed to IR and treated with colcemide for 3 h to arrest cells in metaphase. IR treatment led to a significantly higher percentage of chromosome aberrations in SIAH2-depleted cells than control cells (Supplementary Figure S3G). The percentage of IR-induced chromosome abnormalities in SIAH2-depleted cells was greatly reduced when Flag-SIAH2 WT, but not Flag-SIAH2 ΔSBD, was complemented in SIAH2-depleted cells (Figure 4H), suggesting a critical role for the SBD domain of SIAH2 in chromosome stability.

### Interaction between SIAH2 and CtIP are required to promote end resection

**Figure 5.**
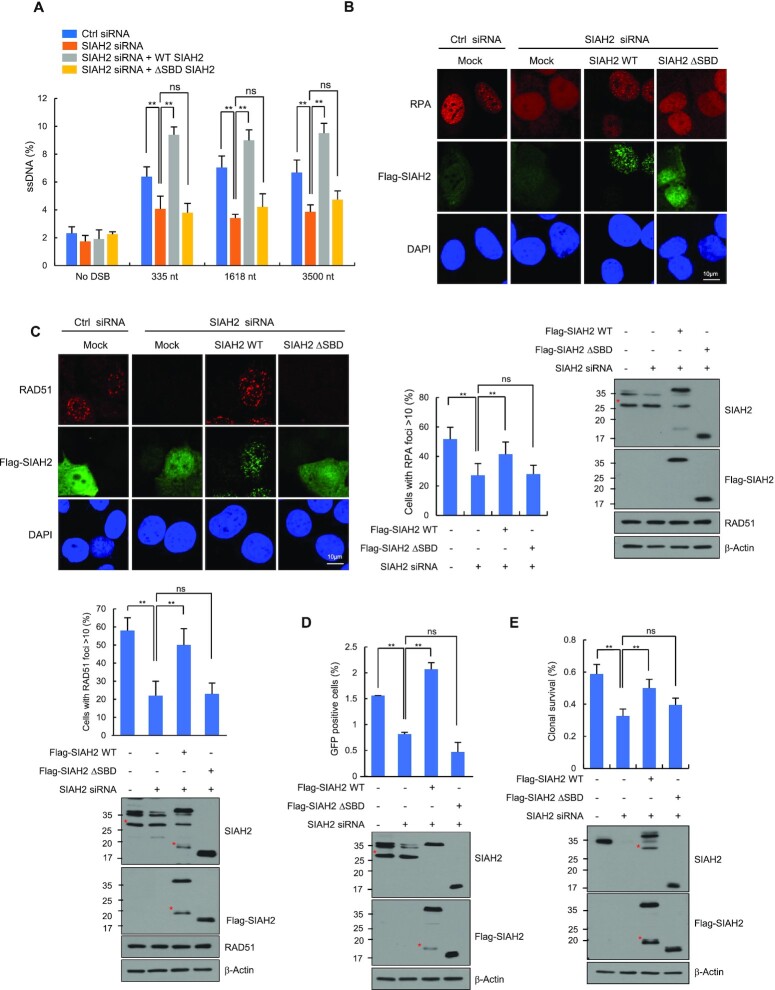
SIAH2 facilitates DNA end resection through CtIP. (**A**) Quantification of ssDNA generated by 5’ end resection at three AsiSI-induced DSBs in either control or SIAH2-depleted AsiSI-ER-U2OS cells reconstituted with Flag-Mock, Flag-SIAH2 WT or Flag-SIAH2 ΔSBD. Results are shown as the mean ± SD (*n* = 3), **P < 0.01. ns, nonsignificant, two-tailed Student's *t*-test. (B and C) Control or SIAH2-depleted HeLa cells reconstituted with Flag-Mock, Flag-SIAH2 WT or Flag-SIAH2 ΔSBD were exposed to 5 Gy IR, fixed at 3 h, and immunostained with anti-RPA and anti-Flag antibodies (**B**) or anti-RAD51 and anti-Flag antibodies (**C**). DNA was stained with DAPI. The percentage of cells containing >10 RPA or RAD51 foci was calculated. Representative images and quantification of RAD51 and RPA foci are shown. Results are shown as the mean ± SD (*n* = 3), **P < 0.01. ns, nonsignificant, two-tailed Student's *t*-test. Asterisks indicate nonspecific bands. (**D**) The efficiency of HR repair in control or SIAH2-depleted DR-GFP-U2OS cells reconstituted with Flag-Mock, Flag-SIAH2 WT or Flag-SIAH2 ΔSBD. Results are shown as the mean ± SD (*n* = 3), **P < 0.01. ns, nonsignificant, two-tailed Student's *t*-test. Asterisks indicate nonspecific bands. (**E**) Clonogenic survival assay of the same cells described in (B). Cells were treated with 2 Gy IR, and colonies were allowed to form over ∼14 days. Results are shown as the mean ± SD (*n* = 3), **P < 0.01. ns, nonsignificant, two-tailed Student's *t*-test. Asterisks indicate nonspecific bands.

Given that SIAH2 promotes IR-induced CtIP ubiquitination via interactions with CtIP, lack of this interaction is expected to cause defects in DNA end resection and HR. To test this hypothesis, we ectopically expressed FLAG-SIAH2 WT and FLAG-SIAH2 ΔSBD in SIAH2-depleted AsiSI-ER-U2OS cells (47) and treated the cells with 4-OHT to induce the nuclear localization of the nuclease. While overexpression of Flag-SIAH2 WT fully rescued the amount of ssDNA generated at all three positions from the AsiSI break sites in SIAH2-depleted cells, deletion of the SBD domain of SIAH2 (Flag-SIAH2 ΔSBD), which completely abrogated its binding to CtIP, failed to do so (Figure 5A). Expression levels of FLAG-SIAH2 ΔSBD and FLAG-SIAH2 WT in the cell were comparable (Supplementary Figure S3H), supporting these conclusions. We then examined the effect of FLAG-SIAH2 ΔSBD on IR-induced RPA and RAD51 foci formation. Introduction of SIAH2 WT reversed impaired RPA and RAD51 recruitment in SIAH2-depleted cells. In contrast, SIAH2 ΔSBD failed to restore IR-induced foci formation of both RPA (Figure 5B) and RAD51 (Figure 5C). As predicted, the defect in HR caused by the depletion of endogenous SIAH2 was largely restored by the addition of SIAH2 WT but not SIAH2 ΔSBD (Figure 5D). In addition, cell survival assays revealed that expression of SIAH2 WT, but not SIAH2 ΔSBD, rescued cell viability in response to IR in SIAH2-depleted cells (Figure 5E). Although these assays were not done with more specific mutations that disrupt the SIAH2-CtIP interaction, these results emphasize the importance of the SBD domain in supporting the DNA end resection and HR repair.

### Ubiquitination of CtIP at five lysine residues in the amino-terminal region is important for end resection and HR

Recent mass spectrometric studies have identified several potential ubiquitination sites in CtIP using the UbiSite or SEPTM strategy (52,53). Among them, five lysines (5K) in amino-terminal CtIP, including K62, K78, K115, K132 and K133, were ubiquitinated by RNF138 in response to IR treatment (33). To determine the sites that are ubiquitinated in response to IR and regulated by SIAH2, we generated CtIP mutants with lysine to arginine mutations either at the five residues in the amino-terminus (CtIP-5KR) or at six residues in the carboxy-terminus, K585, K604, K613, K640, K760 and K782 (CtIP-6KR) as a control (Supplementary Figure S4A). As expected (33), there was much less IR-induced ubiquitination of CtIP-5KR than either WT CtIP or CtIP-6KR (Supplementary Figure S4B). Of note, when CtIP-depleted cells were co-transfected with FLAG-CtIP-5KR, with or without SIAH2 overexpression, SIAH2 expression had little effect on ubiquitination of the CtIP-5KR mutant (Supplementary Figure S4C). Knockdown of SIAH2 also had little effect on ubiquitination of CtIP-5KR mutant (Figure S4D). On the other hand, BRCA1 or RNF138 knockdown reduced CtIP-5KR ubiquitination (Supplementary Figure S4D). Of notice, SIAH2 knockdown or SIAH2 overexpression decreased or increased the ubiquitination level of CtIP-6KR, respectively (Supplementary Figure S4E and S4F). These results suggest that the five amino-terminal lysines of CtIP are the key ubiquitination sites targeted by SIAH2 in response to IR treatment.

**Figure 6.**
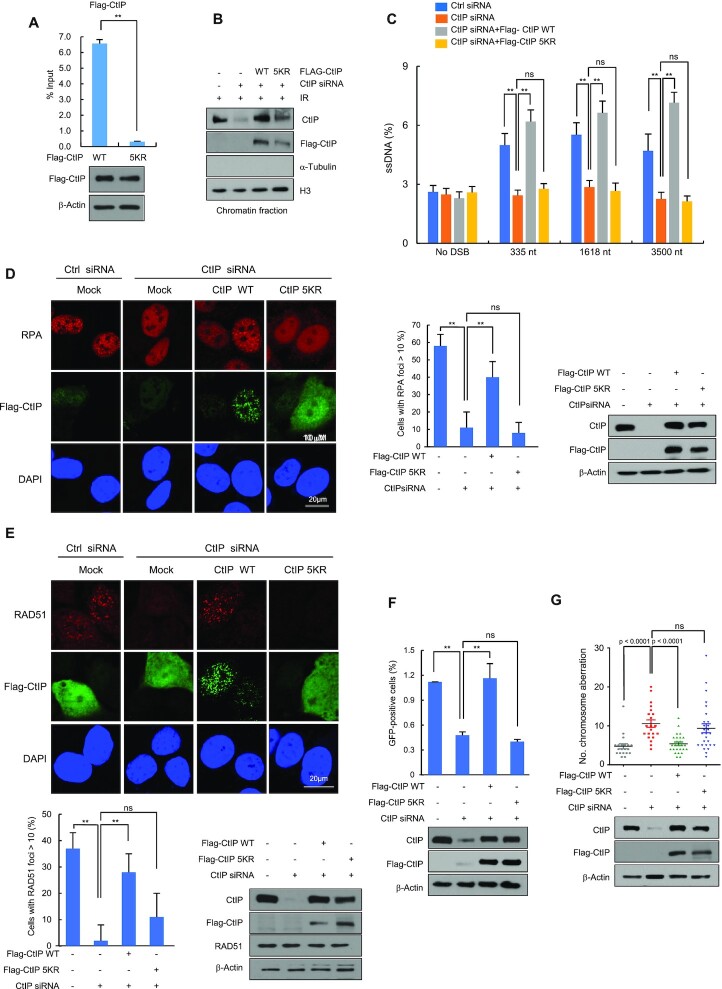
SIAH2 mediates CtIP ubiquitination at K62, K78, K115, K132 and K133, which is important for resection and HR. (**A**) DR-GFP U2OS cells expressing either Flag-CtIP WT or Flag-CtIP-5KR were transfected with an I-SceI expression plasmid for up to 8 h. ChIP experiments were performed using an anti-Flag antibody. Results are shown as the mean ± SD (*n* = 3), **P < 0.01, **P < 0.01, two-tailed Student's *t*-test. (**B**) Control HeLa cells or CtIP-depleted HeLa cells reconstituted with Flag-Mock, Flag-CtIP WT or Flag-CtIP-5KR were treated with 5 Gy IR for 3 h. Chromatin fractions were subjected to western blotting with the indicated antibodies. (**C**) Quantification of ssDNA generated by 5’ end resection at three AsiSI-induced DSBs in either control or CtIP-depleted AsiSI-ER-U2OS cells reconstituted with Flag-Mock, Flag-CtIP WT or Flag-CtIP-5KR. Results are shown as the mean ± SD (*n* = 3), **P < 0.01. ns, nonsignificant, two-tailed Student's *t*-test. (D and E) Control HeLa cells or CtIP-depleted HeLa cells reconstituted with Flag-CtIP WT or Flag-CtIP-5KR were exposed to 5 Gy IR, fixed after 3 h, and immunostained with anti-RPA and anti-Flag antibodies (**D**) or anti-RAD51 and anti-Flag antibodies (**E**). DNA was stained with DAPI. Representative images and the percentage of cells containing >10 foci are shown. Results are shown as the mean ± SD (*n* = 3), **P < 0.01. ns, nonsignificant, two-tailed Student's *t*-test. (**F**) The efficiency of HR repair in either control or CtIP-depleted DR-GFP-U2OS cells reconstituted with Flag-Mock, Flag-CtIP WT or Flag-CtIP-5KR. Results are shown as the mean ± SD (n = 3), **P < 0.01. ns, nonsignificant, two-tailed Student's *t*-test. (**G**) IR-induced chromosomal aberrations were measured in the same cells described in (**B**) 24 h after treatment with 2 Gy of IR. Dot plot shows the number of chromosomal aberrations per cells. *P* values were calculated by the Mann–Whitney test. ns, nonsignificant.

Next, we tested whether ubiquitination of the amino-terminal 5K of CtIP promotes recruitment to DSBs. ChIP-qPCR assays showed that upon I-SceI expression, enrichment of the CtIP-5KR at the DSB sites was ∼12-fold reduced compared to WT CtIP (Figure 6A). Consistently, the 5KR mutant is much less enriched at the chromatin fraction than the WT CtIP (Figure 6B). To examine whether the CtIP-5KR mutation also leads to defects in DNA end resection and HR repair, we transfected either Flag-CtIP WT or Flag-CtIP-5KR into AsiSI-ER-U2OS cells with endogenous CtIP knocked down, and then measured ssDNA upon 4-OHT treatment. We found that ectopic expression of CtIP WT, but not CtIP-5KR, rescued normal ssDNA production (Figure 6C and Supplementary Figure S4G). CtIP-5KR also failed to rescue both IR-induced RPA and RAD51 foci formation and HR in CtIP-depleted cells (Figure 6D–F). Furthermore, the number of chromosomal aberrations was higher in CtIP-depleted cells, a phenotype that could not be rescued by introducing CtIP-5KR (Figure 6G). Taken altogether, these results suggest that modification of CtIP at residues K62, K78, K115, K132 and K133 by ubiquitination is required to facilitate DNA end resection and HR repair.

### SIAH2 promotes CtIP accumulation at replication forks and recovery of stalled forks during replication stress

**Figure 7.**
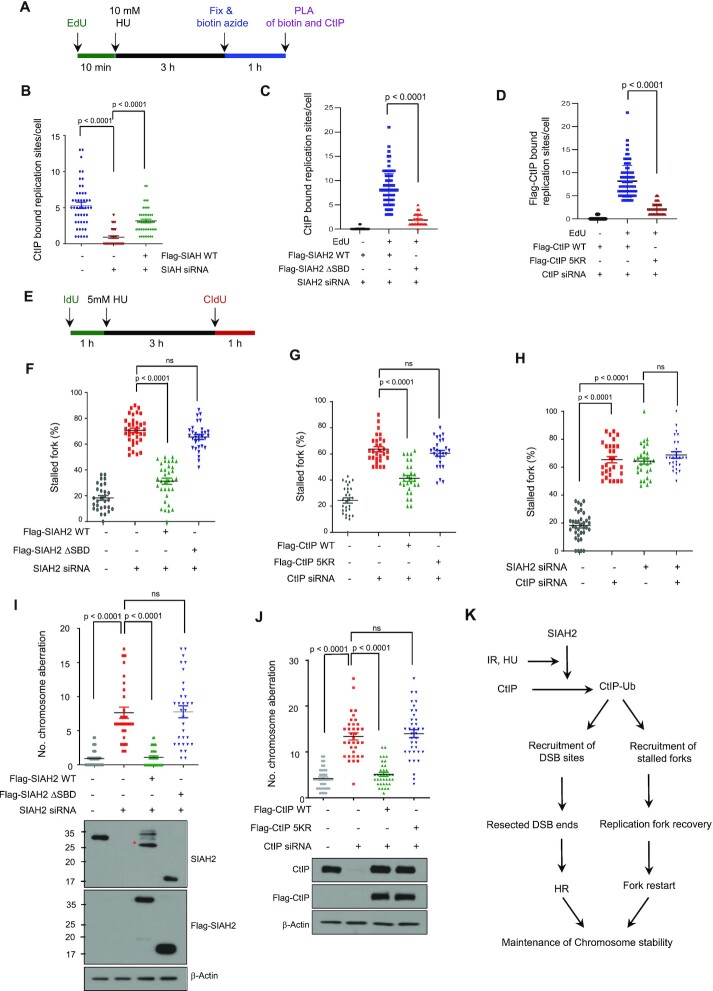
SIAH2 promotes CtIP accumulation at replication forks and promotes recovery of stalled forks. (**A**) Experimental scheme of the SIRF assay to measure interactions between CtIP and nascent DNA in single cells. (**B**) Control HeLa cells, SIAH2-depleted HeLa cells, and SIAH2-depleted HeLa cells reconstituted with Flag-SIAH2 were pulse labeled with EdU for 10 min, followed by 10 mM HU treatment for 3 h. Localization of CtIP at the nascent forks was detected through the SIRF assay. Data represent mean ± SD (*n* = 3). *P* values between indicated samples were calculated using a Mann–Whitney test. (C and D) Localization of CtIP at the nascent forks, measured using the assay described in (B), in SIAH2-depleted HeLa cells reconstituted with either Flag-SIAH2 WT or Flag-SIAH2 ΔSBD (**C**) and CtIP-depleted HeLa cells reconstituted with either Flag-CtIP WT or Flag-CtIP-5KR (**D**). Data represent mean ± SD (*n* = 3). *P* values between indicated samples were calculated using a Mann–Whitney test. (**E**) Schematic for labelling HeLa cells with CIdU and IdU. (F–H) Double-labeled DNA fibers in control HeLa cells or SIAH2-depleted HeLa cells reconstituted with Flag-Mock, Flag-SIAH2 WT or Flag-SIAH2 ΔSBD (**F**), control HeLa cells or CtIP-depleted HeLa cells reconstituted with Flag-Mock, Flag-CtIP WT or Flag-CtIP-5KR (**G**) and control HeLa cells or HeLa cells transfected with the indicated siRNA combinations (**H**) to quantify stalled replication forks. Data are presented as means ± SD (*n* = 3). *P* values between indicated samples were calculated using a Mann–Whitney test. (**I** and **J**) Analysis of chromosome aberrations was carried out with SIAH2-depleted HeLa cells reconstituted with Flag-Mock, Flag-SIAH2 WT or Flag-SIAH2 ΔSBD (**I**) and CtIP-depleted HeLa cells reconstituted with Flag-Mock, Flag-CtIP WT or Flag-CtIP-5KR (**J**) treated with 2 mM HU for 24 h. Dot plot shows the number of chromosome aberrations per cells. Data represent mean ± SD (*n* = 3), **P < 0.01. ns, nonsignificant. *P* values between indicated samples were calculated using a Mann–Whitney test. Asterisk indicates nonspecific band. (**K**) Schematic depicting the role of SIAH2 in CtIP function in end resection and replication fork recovery. See text for details.

In addition to its canonical role in DSB repair, CtIP has roles at the stressed replication forks. For instance, CtIP is recruited by FANCD2 to facilitate fork restart during the stress recovery (18). Indeed, CtIP is found at nascent replication forks by a proteomic screen (21). A role for SIAH2 in recovery of stalled forks was evidenced by the observation that interaction between CtIP and SIAH2 was greatly increased upon HU treatment (Figure 1B). Therefore, it is possible that the SIAH2 also regulates this process via relocating CtIP to the forks. To test this, we performed the PLA-based immunofluorescence assay to test if SIAH2 is required for CtIP to be present at the nascent forks (labeled by EdU) (Figure 7A). First, we were able to establish that CtIP is present at forks, consistent with previous reports (21). Under this condition, the CtIP-EdU PLA signals were markedly lower when SIAH2 was depleted (Figure 7B and Supplementary Figure S5A) This effect was rescued by overexpressing SIAH2 WT, but not by ΔSIAH2 SBD (Figure 7C and Supplementary Figure S5B), indicating a critical role for SIAH2 in interacting with CtIP to sites of DNA synthesis upon transient fork stalling. Consistent with the idea that CtIP ubiquitination and recruitment to DSBs are important regulatory steps, the number of Flag-CtIP-5KR-EdU signals was markedly lower than that of Flag-CtIP WT (Figure 7D and Supplementary Figure S5C). These altogether suggest that SIAH2-mediated ubiquitination is important for CtIP localization to replication forks under replication stress conditions.

To determine whether SIAH2 plays a role in replication fork restart, we measured stalled replication forks after HU treatment by pulse-labelling cells with CIdU, treating with HU to induced fork stalling, and then labeling with IdU (Figure 7E). We found that cells depleted in SIAH2 had a higher percentage of stalled replication forks, and that reconstitution of SIAH2-depleted cells with Flag-SIAH2 WT but not Flag-SIAH2 ΔSBD, rescued stalled replication forks (Figure 7F and Supplementary Figure S5D). Consistently, reconstitution of CtIP-depleted cells with Flag-CtIP WT, but not Flag-CtIP-5KR, rescued stalled forks (Figure 7G and Supplementary Figure S5E). To further confirm that the regulatory role of SIAH2 in replication fork protection is dependent on CtIP and not a different pathway, we depleted both SIAH2 and CtIP and did not observe any additive increase in the number of stalled forks fork (Figure 7H and Supplementary Figure S5F). We then investigated the effect of SIAH2 on fork stability in BRCA-depleted cells and found that SIAH2 knockdown in BRCA1-depleted cells further aggravates the degradation of stalled forks (Supplementary Figure S5G), suggesting that SIAH2 contribute to fork stability through a BRCA1-independent pathway.

To obtain further insight into the role of SIAH2 in maintaining chromosome stability under replication stress, we analyzed the effects of SIAH2-mediated CtIP ubiquitination on chromosome aberrations after HU-induced replication stress. SIAH2-depleted cells exhibited higher levels of HU-induced chromosomal aberrations than control cells, and reintroduction of Flag-SIAH2 WT, but not Flag-SIAH2 ΔSBD, reduced chromosome aberrations in SIAH2-depleted cells (Figure 7I). Consistently, the increased number of chromosomal aberrations observed in CtIP-depleted cells was rescued by reconstituting with Flag-CtIP WT but not Flag-CtIP-5KR (Figure 7J). Together, these results indicate that SIAH2-mediated ubiquitination at the five amino-terminal lysine residues (5K) of CtIP plays a crucial role in maintaining chromosome stability after fork stalling.

Lastly, in support of the role of SIAH2 in stressed fork-metabolism, SIAH2-depleted cells were hypersensitive to HU (Supplementary Figure S6A) and accumulated more DNA breaks upon HU treatment as detected through γ-H2AX staining (Supplementary Figure S6B). Moreover, CtIP ubiquitination was stimulated by HU (Supplementary Figure S6C), this effect was significantly reduced in the CtIP-5KR mutant (Supplementary Figure S6D), and SIAH2 expression had no effect on CtIP-5KR ubiquitination (Supplementary Figure S6E).

## DISCUSSION

Herein, we provide evidence that SIAH2 is a new regulator of the HR repair. Through an unbiased screen, we found SIAH2 as an interactor of CtIP. We found that SIAH2 induces a non-proteolytic ubiquitination of CtIP. A CtIP mutant that is less ubiquitinated by SIAH2 (CtIP-5KR) cannot localize efficiently to DSB sites. Importantly, this mutant cannot support the normal HR repair activities. Consistently, depletion of SIAH2 compromises the CtIP localization to DSBs, end resection, and overall HR repair capacity. Thus, we have identified a new protein network, the SIAH2-CtIP axis, in regulation of the HR repair. We further found that depletion of SIAH2 or expressing the CtIP-5KR mutant reduces the replication fork recovery under replication stress, consistent with the recently recognized roles of HR repair in the replication fork integrity maintenance.

SIAH2 is a new addition to the growing list of CtIP modifiers. CtIP is regulated by SUMOylation via CBX4 and PIAS4 (23,30). ATM, ATR and cyclin-dependent kinases (CDKs) phosphorylate CtIP and the phosphorylation promotes DNA end resection (54–56). Ubiquitination of CtIP has been also reported; BRCA1 (29) and RNF138 (33) induce a non-proteolytic ubiquitination of CtIP, which in turn regulates the HR repair and the G2/M checkpoint control. SIAH1, a close homologue of SIAH2, induces polyubiquitination of CtIP, which leads to proteasomal degradation (51). The latter finding suggests that SIAH1 is a negative regulator of HR repair (46), while our work suggests that SIAH2 is a positive regulator of HR repair. It is notable that two closely related proteins impose an antagonistic regulation on the same target protein. It is interesting to speculate that these proteins may be activated in a temporal manner (for example, SIAH2 is needed to activate the HR repair while SIAH1 turns it off later), to facilitate the completion of HR repair in a coordinated manner. In this regard, it is interesting that p53 induces expression of both SIAH1 and SIAH2 (35,36). Deubiquitination of CtIP by USP52 (32) may be also coordinated in this regard, in overall promoting the HR repair.

While the non-proteolytic polyubiquitination of CtIP by the three E3 ubiquitin ligases, BRCA1, RNF138 and SIAH2, all promote the CtIP function in positive ways, distinct roles of or interplay among these ubiquitination events remain to be elucidated. In particular, the observed roles of SIAH2 in promoting the CtIP recruitment to DSBs herein is seemingly redundant with the reported role of RNF138 (33); this study found that the CtIP recruitment is not dependent on BRCA1. As our study found that depleting SIAH2 or RNF138 similarly affect CtIP ubiquitination (Supplementary Figure S2F), it is reasonable to speculate that there is a functional interplay between SIAH2 and RNF138 in promoting the CtIP ubiquitination and subsequent recruitment to DSBs. Our initial attempts so far did not yet reveal such interplay; depleting SIAH2 did not alter the protein level or chromatin retention of RNF138, or vice versa (Figure 2B and Supplementary Figure S7). We also could not observe detectable physical interaction between the two ligases (data not shown). It is also worth speculating that the polyubiquitination occurs in a collaborative manner, where the initial ubiquitination by one ligase is followed by subsequent extension or editing by another ligase, in a manner analogous to the chain extension by an E4 ligase (57). Alternatively, these two ligases may regulate CtIP in different times in a context-dependent manner, although this scenario is unlikely as both studies used the IR-induced DSBs as a source for DNA damage. We additionally showed that the SIAH2-mediated CtIP regulation is required for mitigating the replication fork stress (Figure 7). Whether RNF138 also regulates CtIP under this context is unknown. That being said, more thorough future investigation is needed to sort out the different roles of these two E3 ligases in CtIP regulation. Regardless of the differential contributions between SIAH2 and RNF138, our work further highlights the importance of the CtIP ubiquitination (via analyzing the 5K mutant), in positive regulation of the CtIP functions in HR repair.

How does CtIP ubiquitination induce its recruitment to DSBs? This probably is the most intriguing aspect of our finding. One possibility is that ubiquitination induces dimerization of CtIP, which is known to regulate CtIP localization (58). Alternatively, the ubiquitination enhances interaction with its upstream regulators, such as FANCD2 (18,59,60), perhaps in synergism with the existing module that mediates the CtIP-FANCD2 interaction. This situation may be confined to stressed replication forks or ICL repair, where the FANCD2-CtIP interplay is important. Or, the ubiquitination may play roles in interacting with the MRN or BRCA1 (29) during the IR-induced DSB repair. At this point we are only able to speculate that these CtIP-interactors may have cryptic ubiquitin binding domains that increases affinity to ubiquitinated CtIP. Further investigations are under way. Regardless, an important implication of our work is that the recruitment of CtIP is a crucial regulatory step for the correct functioning of CtIP. CtIP has been proposed to have at least two distinct functions at the stressed forks; in promoting the restart of stalled forks (18) and in preventing the degradation of stalled forks (19)—two situations where distinct recovery mechanisms are thought to be employed. Our current study at least suggest that the CtIP-5KR mutant does not support the replication restart (Fig 7), a finding consistent with the idea that the HR repair activity is needed for the fork restart during recovery (61). As CtIP is involved in fork degradation in BRCA1-deficient cells (19), SIAH2 affects the fork stability in these cells, suggesting that SIAH2 mediates fork protection in a BRCA1-independent manner, similar to CtIP. Whether SIAH2 also regulates CtIP’s other functions, such as fork protection and R-loop resolution (62), is currently unknown and needs to be investigated in future.

Based on our findings, we propose the following model (Fig 7K); when DSBs occur or replication forks stall, SIAH2 can regulate the binding of CtIP to the MRN complex by promoting ubiquitination of CtIP at five N-terminal lysine residues. This leads to the recruitment of CtIP to DSB sites and stalled forks, thereby promoting DSB end resection, HR repair, and recovery of stalled replication forks. In all, identification of SIAH2 as a new factor promoting the HR repair and replication fork restart, and a new response factor to DSBs in general, provides a new insight and opportunities in the fields.

## DATA AVAILABILITY

The authors confirm that the data supporting the findings of this study are available within the article and its supplementary materials.

## Supplementary Material

gkac808_Supplemental_File
