# Supplementary material for: SIAH2 regulates DNA end resection and replication fork recovery by promoting CtIP ubiquitination
Source: Nucleic Acids Res. 2022 Sep 26;50(18):10469–86. doi: 10.1093/nar/gkac808 (PMC9561274; doi:10.1093/nar/gkac808)
Supplement: gkac808_Supplemental_File [file gkac808_supplemental_file.pdf]

SIAH2 regulates DNA end resection and replication fork recovery by promoting CtIP ubiquitination

7 supplementary figures

2 supplementary tables

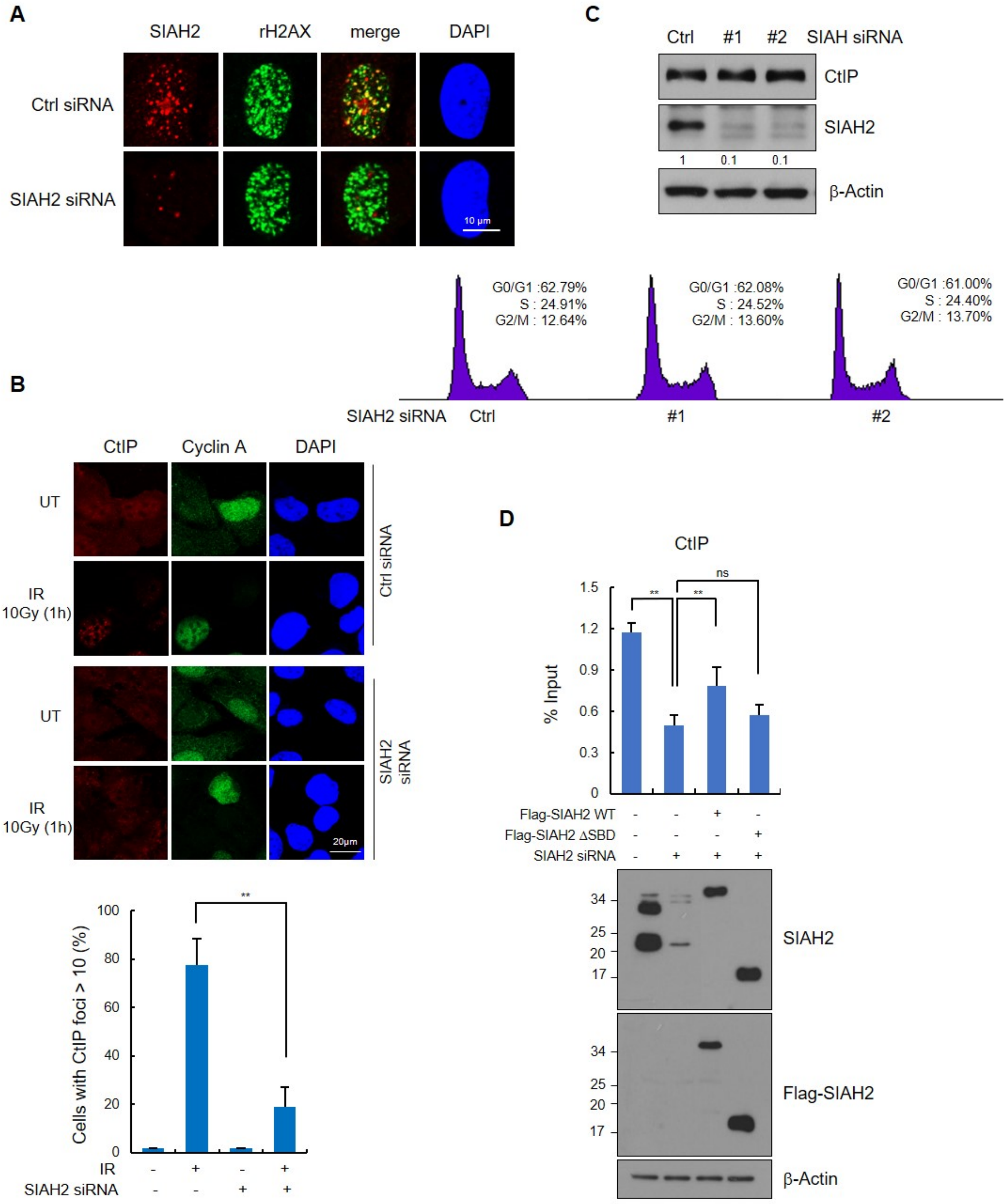

Figure S1. SIAH2 contributes to CtIP recruitment at DSB sites.

(**A**) Control and SIAH2-depleted HeLa cells were treated with 5 Gy IR, fixed at 1 h, and immunostained with anti-SIAH2 antibody. DNA was stained with DAPI. (**B**) Control and SIAH2-

depleted HeLa cells were either untreated or treated with 5 Gy IR, fixed at 1 h, and immunostained with anti-CtIP and anti-Cyclin A (S/G2 marker) antibodies. DNA was stained with DAPI. CtIP foci were analyzed in Cyclin A-positive cells. Representative images and the percentage of cells containing >10 CtIP foci are shown. Data represent mean ± SD (n = 3), ** P < 0.01, two-tailed Student's *t*-test. (**C**) Cell cycle distribution in control and SIAH2 siRNA-transfected HeLa cells. The numbers were relative levels of SIAH2 expression compared to control siRNA-transfected cells. (**D**) Control DR-GFP-U2OS cells, SIAH2-depleted DR-GFP-U2OS cells, and SIAH2 knockdown DR-GFP-U2OS cells reconstituted with Flag-SIAH2 WT or Flag-SIAH2 ΔSBD were transfected with an I-SceI expression plasmid for up to 8 h. ChIP experiments were performed using an anti-CtIP antibody. Whole cell lysates were analyzed by immunoblotting using the indicated antibodies. The numbers were relative levels of SIAH2 expression compared to control siRNA-transfected cells. Data represent mean ± SD (n = 3), ** *P* < 0.01. Statistical analysis was performed with the two-tailed Student's *t*-test.

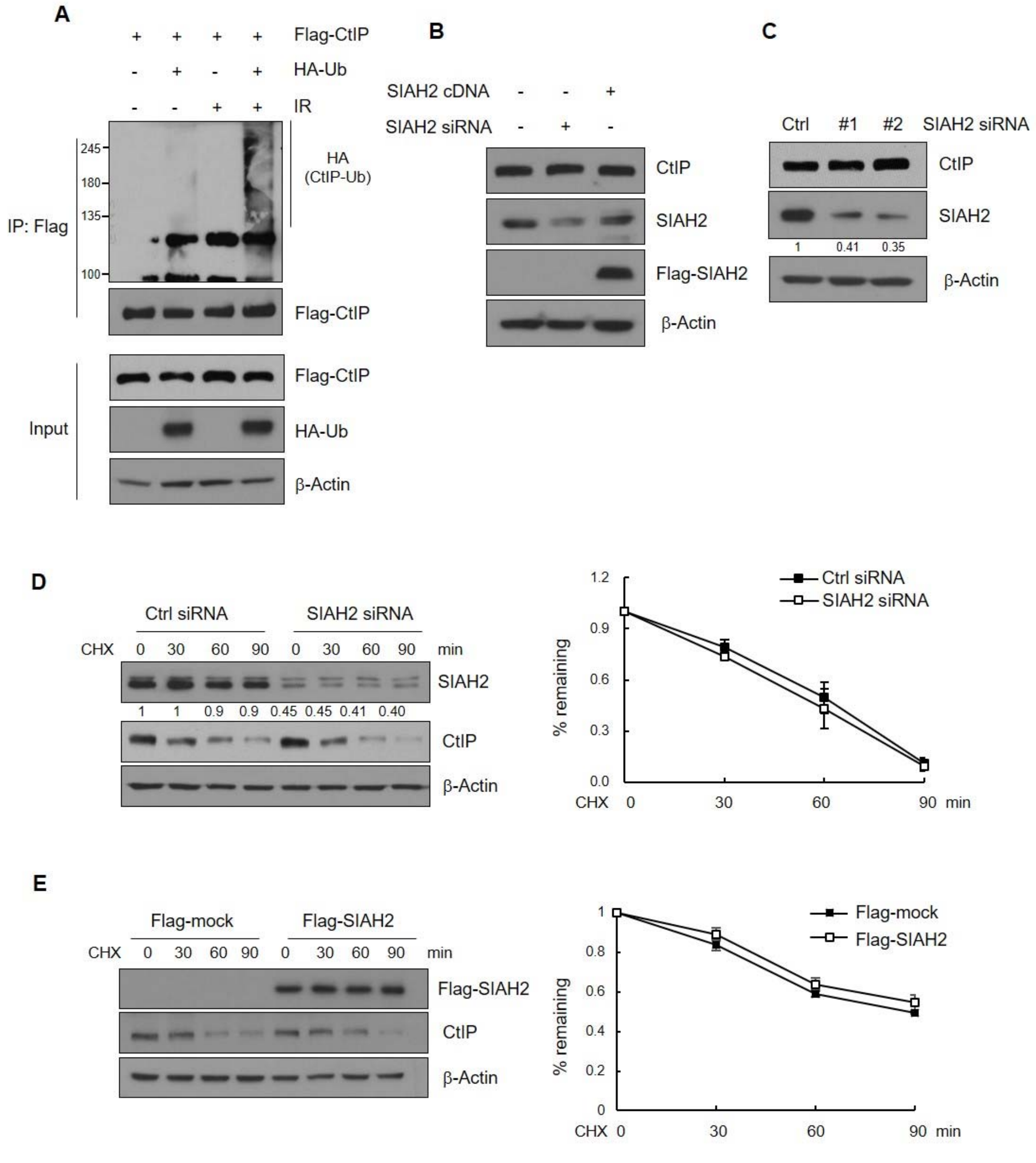
A
Flag-CtIP
HA-Ub
IR
245
180
135
100
HA
(CtIP-Ub)
IP: Flag
Flag-CtIP
Input
Flag-CtIP
HA-Ub
β-Actin
B
SIAH2 cDNA
SIAH2 siRNA
CtIP
SIAH2
Flag-SIAH2
β-Actin
C
Ctrl
#1
#2
SIAH2 siRNA
CtIP
SIAH2
1
0.41
0.35
β-Actin
D
Ctrl siRNA
SIAH2 siRNA
CHX
0 30 60 90 0 30 60 90 min
SIAH2
1 1 0.9 0.9 0.45 0.45 0.41 0.40
CtIP
β-Actin
Ctrl siRNA
SIAH2 siRNA
% remaining
1.2
0.9
0.6
0.3
0.0
CHX 0 30 60 90 min
E
Flag-mock
Flag-SIAH2
CHX
0 30 60 90 0 30 60 90 min
Flag-SIAH2
CtIP
β-Actin
Flag-mock
Flag-SIAH2
% remaining
1
0.8
0.6
0.4
0.2
0
CHX 0 30 60 90 min

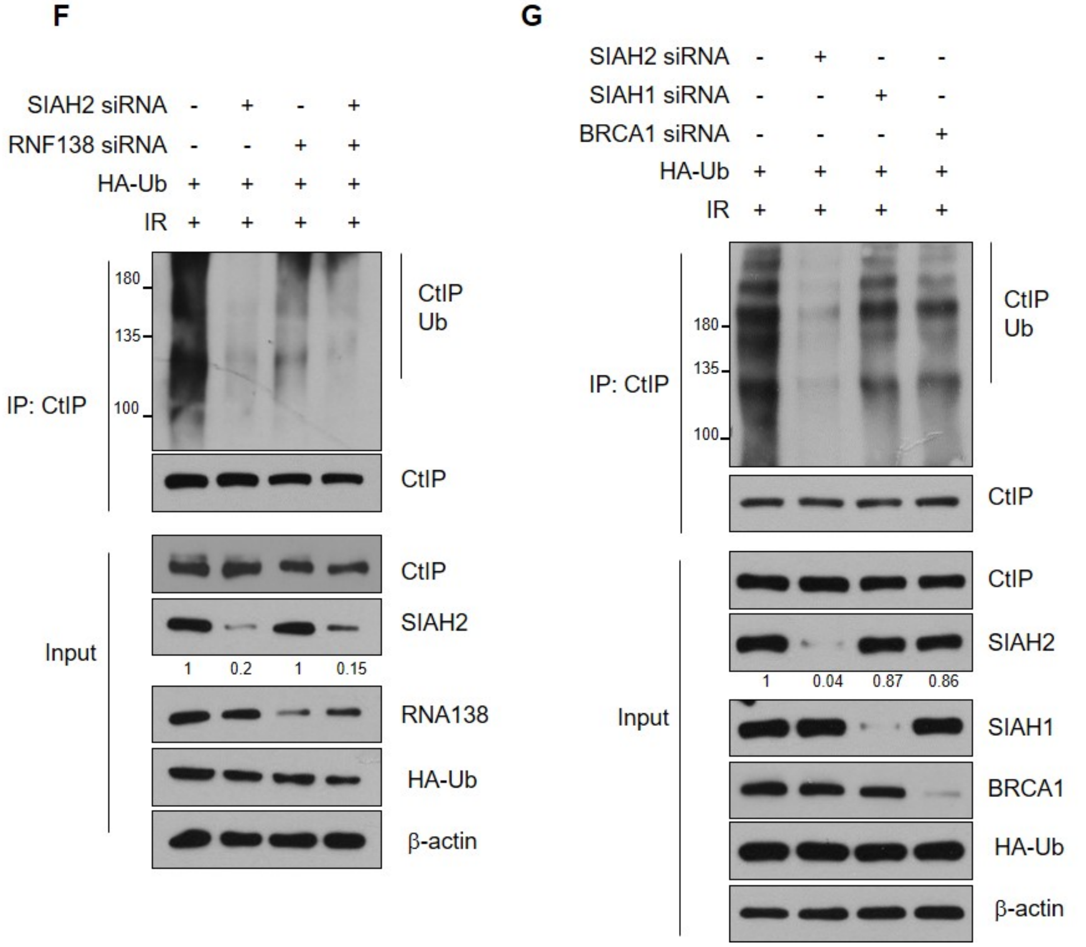

Figure S2. SIAH2 ubiquitinates CtIP.

(**A**) HEK293T cells expressing Flag-CtIP were transfected with or without HA-ubiquitin and were either untreated or treated with 10 Gy of IR for 30 minutes. Total cell lysates were immunoprecipitated with anti-Flag antibody and immunoblotted with the indicated antibodies. (**B**) HeLa cells were transfected with either Flag-SIAH2 or SIAH2 siRNA. 48 h later, total cell lysates were immunoblotted with the indicated antibodies. (**C**) HeLa cells were transfected with either a scrambled negative control siRNA or two different siRNAs against SIAH2 (#1 and #2). 48 h later, total cell lysates were immunoblotted with the indicated antibodies. The numbers were relative levels of SIAH2 expression compared to control siRNA-transfected cells. (**D**) CtIP protein stability 48 h after transfection of cells with either control siRNA or SIAH2 siRNA following cycloheximide treatment (10 μg/ml) for the times indicated. Data are shown as mean ± SD. (**E**)

CtIP protein stability 48 h after transfection of cells with either control vector or Flag-SIAH2 expression vector following cycloheximide treatment (10 μg/ml) for the times indicated. Data are shown as mean ± SD. (**F**) HeLa cells cotransfected with HA-ubiquitin and either a scrambled negative control siRNA, siRNAs against SIAH2 or RNF138, or both SIAH2 and RNF138 siRNAs were treated with 10 Gy IR for 30 minutes. Total cell lysates were immunoprecipitated with anti-CtIP antibody and immunoblotted with the indicated antibodies. (**G**) HeLa cells cotransfected with HA-ubiquitin and either a scrambled negative control siRNA or siRNAs against SIAH2, SIAH1, or BRCA1 were treated with 10 Gy IR for 30 minutes. Total cell lysates were immunoprecipitated with anti-CtIP antibody and immunoblotted with the indicated antibodies.

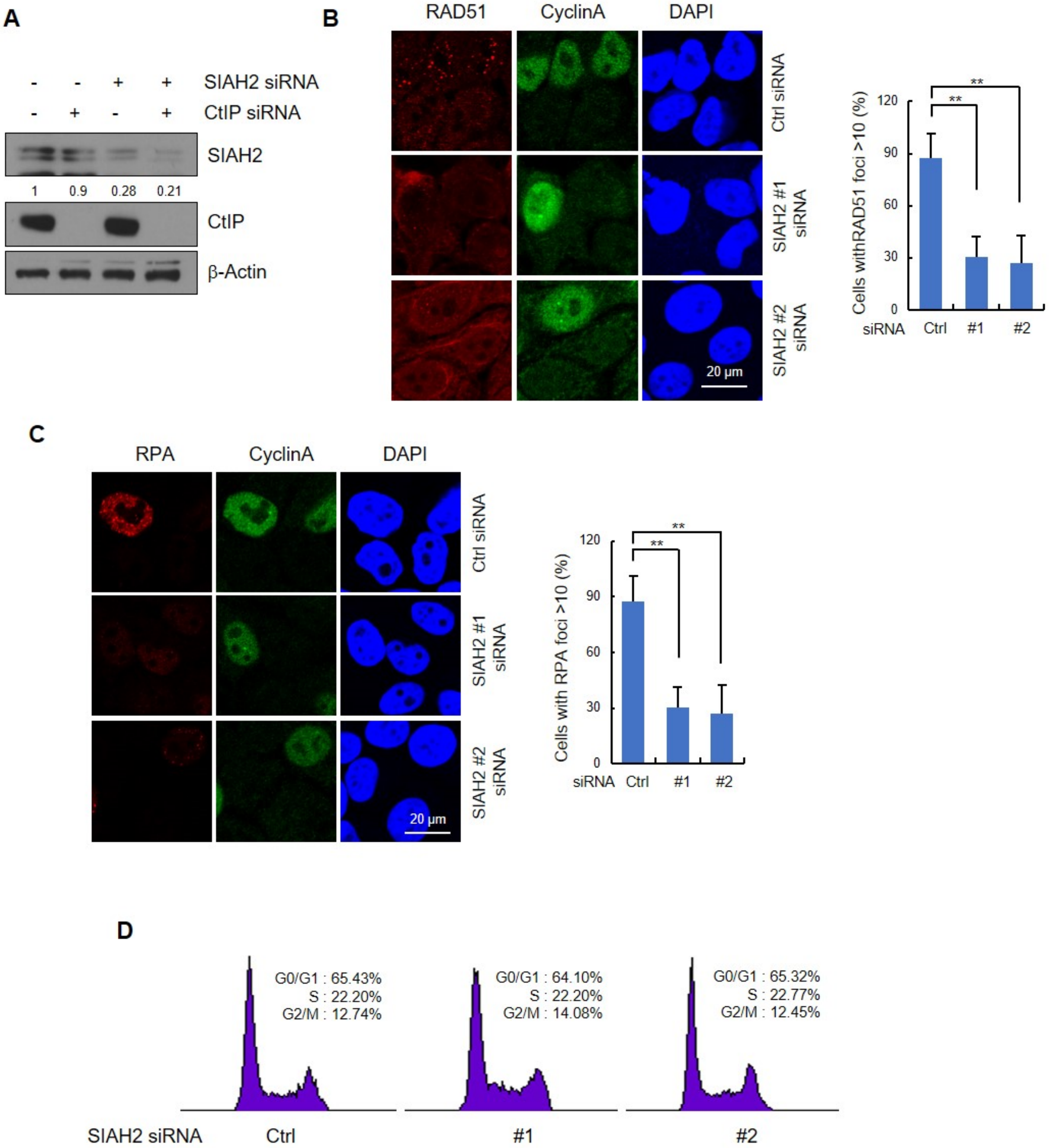
A
SIAH2 siRNA
CtIP siRNA
SIAH2
1 0.9 0.28 0.21
CtIP
β-Actin
B
RAD51
CyclinA
DAPI
Ctrl siRNA
SIAH2 #1 siRNA
SIAH2 #2 siRNA
20 μm
Cells withRAD51 foci >10 (%)
siRNA Ctrl #1 #2
C
RPA
CyclinA
DAPI
Ctrl siRNA
SIAH2 #1 siRNA
SIAH2 #2 siRNA
20 μm
Cells with RPA foci >10 (%)
siRNA Ctrl #1 #2
D
G0/G1 : 65.43%
S : 22.20%
G2/M : 12.74%
G0/G1 : 64.10%
S : 22.20%
G2/M : 14.08%
G0/G1 : 65.32%
S : 22.77%
G2/M : 12.45%
SIAH2 siRNA Ctrl #1 #2

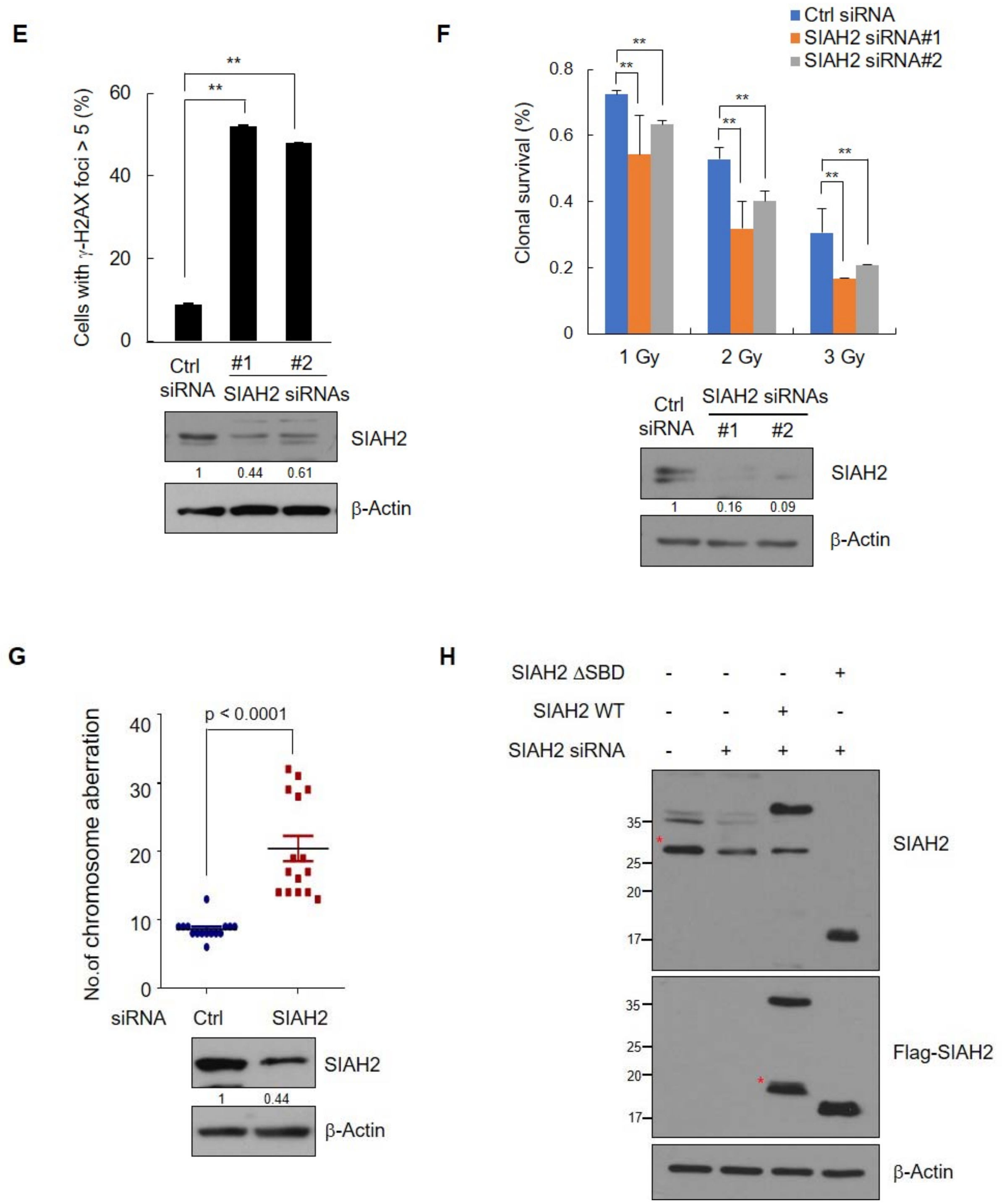

Figure S3. SIAH2 maintains genomic stability.

(**A**) The expression levels of SIAH2 and CtIP in AsiSI-ER-U2OS cells transfected with either control siRNA or siRNAs against CtIP, SIAH2, or both SIAH2 and CtIP. The numbers were

relative levels of SIAH2 expression compared to control siRNA-transfected cells. **(B, C)** HeLa cells transfected with either control siRNA or two different SIAH2-target siRNAs were treated with or without 5 Gy IR, fixed at 3 h, and immunostained with anti-RAD51 and anti-Cyclin A antibodies (**B**) or anti-RPA and anti-Cyclin A antibodies (**C**). DNA was stained with DAPI. RAD51 and RPA foci were analyzed in Cyclin A-positive cells. The percentage of cells containing > 10 foci was calculated. Representative images and quantification of RAD51 and RPA foci are shown. Whole cell lysates were analyzed by immunoblotting using the indicated antibodies. Data represent mean ± SD (n = 3), ** $P < 0.01$, two-tailed Student's *t*-test. **(D)** Cell cycle distribution in control and SIAH2 siRNA-transfected DR-GFP-U2OS cells. The numbers were relative levels of SIAH2 expression compared to control siRNA-transfected cells. **(E)** Control- and SIAH2-depleted HeLa cells were treated with 5 Gy of IR. Cells were fixed at 24 h after IR irradiation and immunostained for γ-H2AX. The percentage of cells with > 5 foci for γ-H2AX is shown. The numbers were relative levels of SIAH2 expression compared to control siRNA-transfected cells. Data represent mean ± SD (n = 3), ** $P < 0.01$. Statistical analysis was performed with the two-tailed Student's *t*-test. (**F**) Colony forming ability of control and SIAH2 knockdown HeLa cells treated with the indicated doses of IR. The numbers were relative levels of SIAH2 expression compared to control siRNA-transfected cells. Survival data is presented as mean ± SD (n = 3). ** $P < 0.01$. Statistical analysis was performed with the two-tailed Student's *t*-test. (**G**) Quantification of chromosomal aberrations in control and SIAH2 knockdown HeLa cells following treatment with 2 Gy IR for 24 h. The numbers were relative levels of SIAH2 expression compared to control siRNA-transfected cells. Data represent mean ± SD (n = 3). *P* values between indicated samples were calculated using a Mann-Whitney test. **(H)** The expression levels of endogenous SIAH2 and Flag-SIAH2 in either control or SIAH2-depleted AsiSI-ER-U2OS cells reconstituted with Flag-Mock, Flag-SIAH2 WT, or Flag-SIAH2 ΔSBD. Asterisks indicate nonspecific bands.

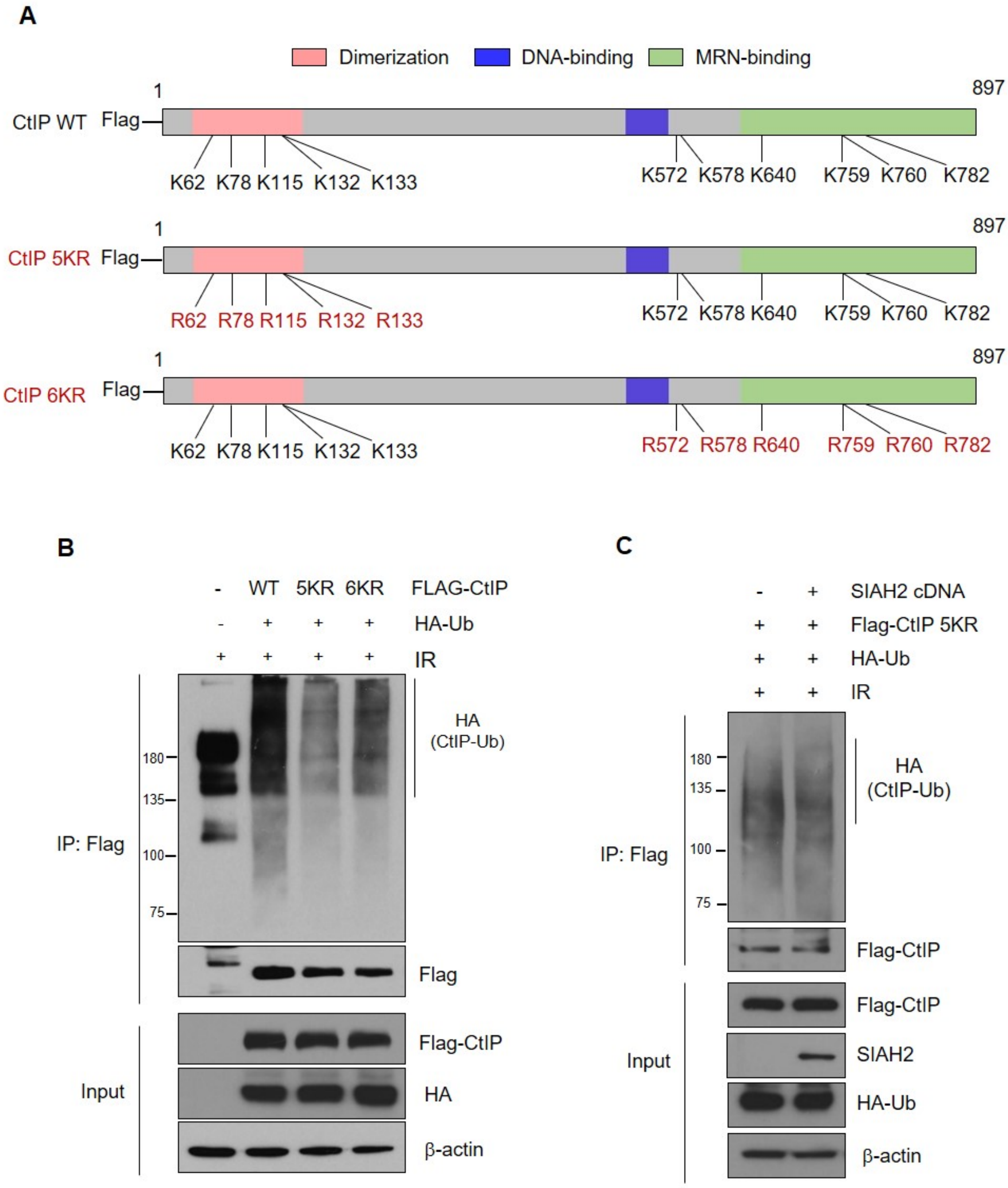
A
Dimerization
DNA-binding
MRN-binding
1
897
CtIP WT
Flag
K62 K78 K115 K132 K133
K572 K578 K640
K759 K760 K782
CtIP 5KR
R62 R78 R115 R132 R133
CtIP 6KR
R572 R578 R640
R759 R760 R782
B
- WT 5KR 6KR FLAG-CtIP
- + + + HA-Ub
+ + + + IR
IP: Flag
180
135
100
75
HA
(CtIP-Ub)
Flag
Input
Flag-CtIP
HA
β-actin
C
- + SIAH2 cDNA
+ + Flag-CtIP 5KR
+ + HA-Ub
+ + IR
IP: Flag
Flag-CtIP
SIAH2
HA-Ub

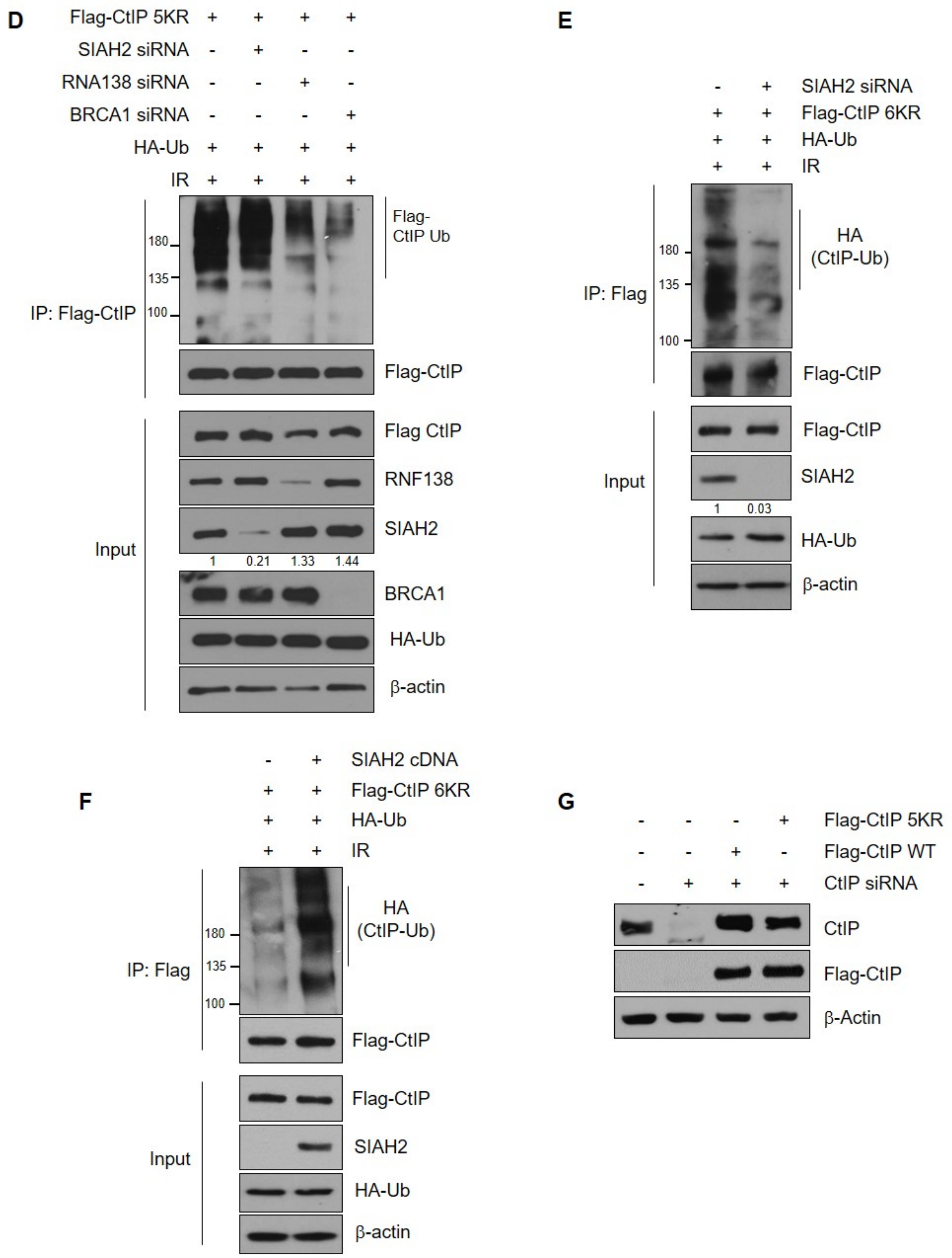

Figure S4. CtIP is ubiquitinated at five N-terminal lysine residues in response to IR treatment.

(**A**) Schematic diagram of CtIP domain structure of wild-type (WT) CtIP and relevant ubiquitination sites in this study, along with substituted in the corresponding site mutants of Flag-tagged CtIP. (**B**) Control HEK293T cells or HEK293T cells transfected with HA-ubiquitin along with Flag-CtIP WT, Flag-CtIP-5KR, or Flag-CtIP-6KR were treated with 10 Gy IR for 30 minutes. Total cell lysates were immunoprecipitated with anti-Flag antibody and immunoblotted with the indicated antibodies. (**C**) Flag-CtIP-5KR-expressing HEK293T cells were transfected with HA-ubiquitin with or without SIAH2 and treated with 10 Gy IR for 30 minutes. Total cell lysates were immunoprecipitated with anti-Flag antibody and immunoblotted with the indicated antibodies.
(**D**) Flag-CtIP-5KR-expressing HEK293T cells were transfected with HA-ubiquitin with indicated siRNs and treated with 10 Gy IR for 30 minutes. Total cell lysates were immunoprecipitated with anti-Flag antibody and immunoblotted with the indicated antibodies. (**E**) Flag-CtIP-6KR-expressing HEK293T cells were transfected with HA-ubiquitin with control siRNA or SIAH2 siRNA were treated with 10 Gy IR for 30 minutes. Total cell lysates were immunoprecipitated with anti-Flag antibody and immunoblotted with the indicated antibodies. (**F**) Flag-CtIP-6KR-expressing HEK293T cells were transfected with HA-ubiquitin with or without SIAH2 and treated with 10 Gy IR for 30 minutes. Total cell lysates were immunoprecipitated with anti-Flag antibody and immunoblotted with the indicated antibodies. (**G**) The expression levels of endogenous CtIP and Flag-CtIP in either control or CtIP-depleted AsiSI-ER-U2OS cells reconstituted with Flag-Mock, Flag-CtIP WT, or Flag-CtIP-5KR.

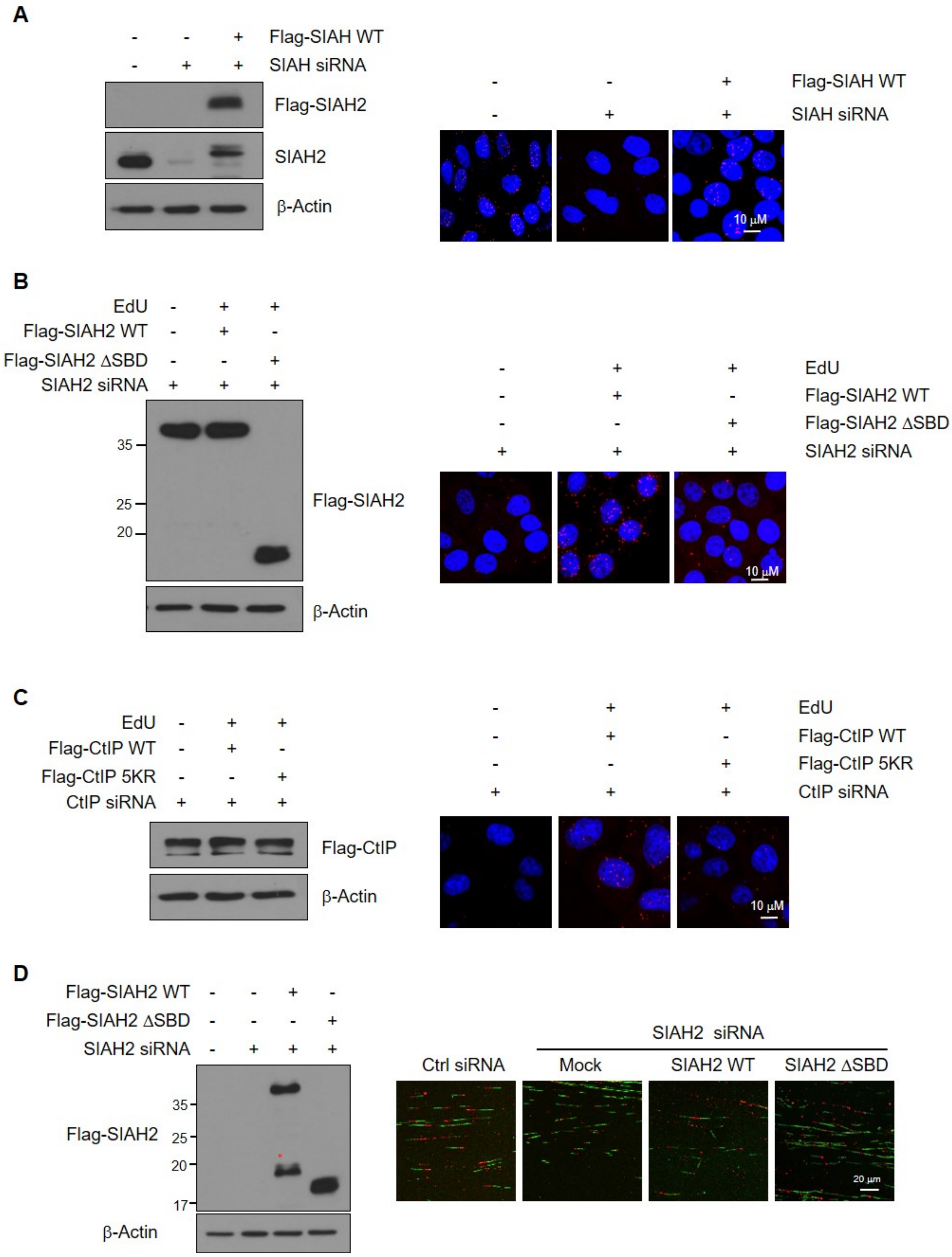
A
Flag-SIAH WT
SIAH siRNA
Flag-SIAH2
SIAH2
β-Actin
Flag-SIAH WT
SIAH siRNA
10 μM
B
EdU
Flag-SIAH2 WT
Flag-SIAH2 ΔSBD
SIAH2 siRNA
35
25
20
Flag-SIAH2
β-Actin
EdU
Flag-SIAH2 WT
Flag-SIAH2 ΔSBD
SIAH2 siRNA
10 μM
C
EdU
Flag-CtIP WT
Flag-CtIP 5KR
CtIP siRNA
Flag-CtIP
β-Actin
EdU
Flag-CtIP WT
Flag-CtIP 5KR
CtIP siRNA
10 μM
D
Flag-SIAH2 WT
Flag-SIAH2 ΔSBD
SIAH2 siRNA
Flag-SIAH2
35
25
20
17
β-Actin
SIAH2 siRNA
Ctrl siRNA
Mock
SIAH2 WT
SIAH2 ΔSBD
20 μm

E

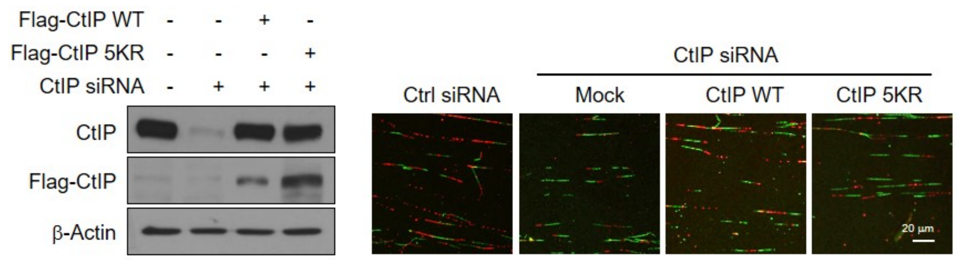

F

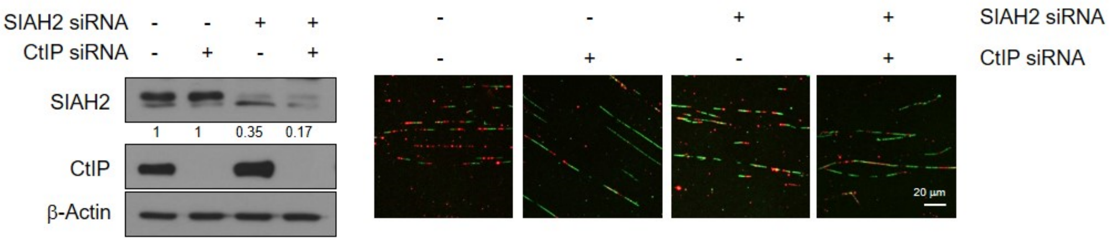

G

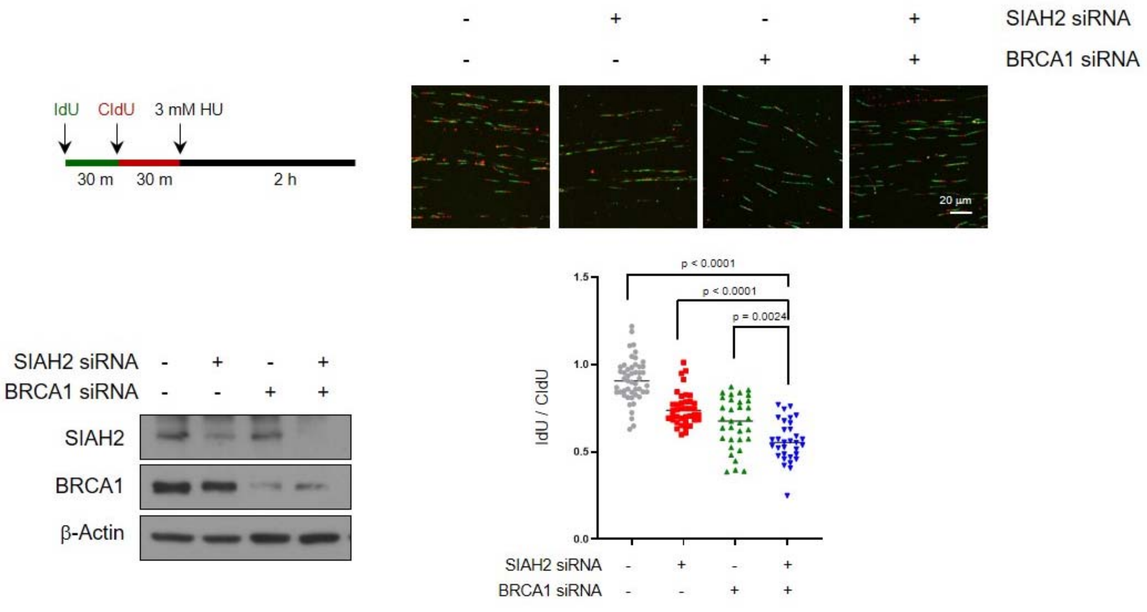

Figure S5. SIAH2 contributes to replication fork restart through CtIP.

(**A**) Control HeLa cells, SIAH2-depleted HeLa cells, and SIAH2-depleted HeLa cells reconstituted with Flag-SIAH2 were pulse labeled with EdU for 10 min, followed by 10 mM HU treatment for

3 h. Localization of CtIP at the nascent forks was detected through the SIRF assay. Whole cell lysates were analyzed by western blotting using the indicated antibodies. Representative images are shown. (**B** and **C**) Localization of CtIP at the nascent forks, measured using the assay described in (**A**), in SIAH2-depleted HeLa cells reconstituted with either Flag-SIAH2 WT or Flag-SIAH2 ΔSBD (**B**) and CtIP-depleted HeLa cells reconstituted with either Flag-CtIP WT or Flag-CtIP-5KR (**C**). Whole cell lysates were analyzed by western blotting using the indicated antibodies. Representative images are shown. (**D-F**) Double-labeled DNA fibers in control HeLa cells or SIAH2-depleted HeLa cells reconstituted with Flag-Mock, Flag-SIAH2 WT, or Flag-SIAH2 ΔSBD (**D**), control HeLa cells or CtIP-depleted HeLa cells reconstituted with Flag-Mock, Flag-CtIP WT, or Flag-CtIP-5KR (**E**), and control HeLa cells or HeLa cells transfected with the indicated siRNA combinations (**F**) to quantify stalled replication forks. Whole cell lysates were analyzed by western blotting using the indicated antibodies. Representative images are shown. (**G**) Double-labeled DNA fibers in control, SIAH2-depleted, BRCA1-depleted, and SIAH2 and BRCA1-co-depleted HeLa cells to quantify fork degradation. Whole cell lysates were analyzed by western blotting using the indicated antibodies. Representative images are shown. Data are presented as means ± SD (n = 3). *P* values between indicated samples were calculated using a Mann-Whitney test.

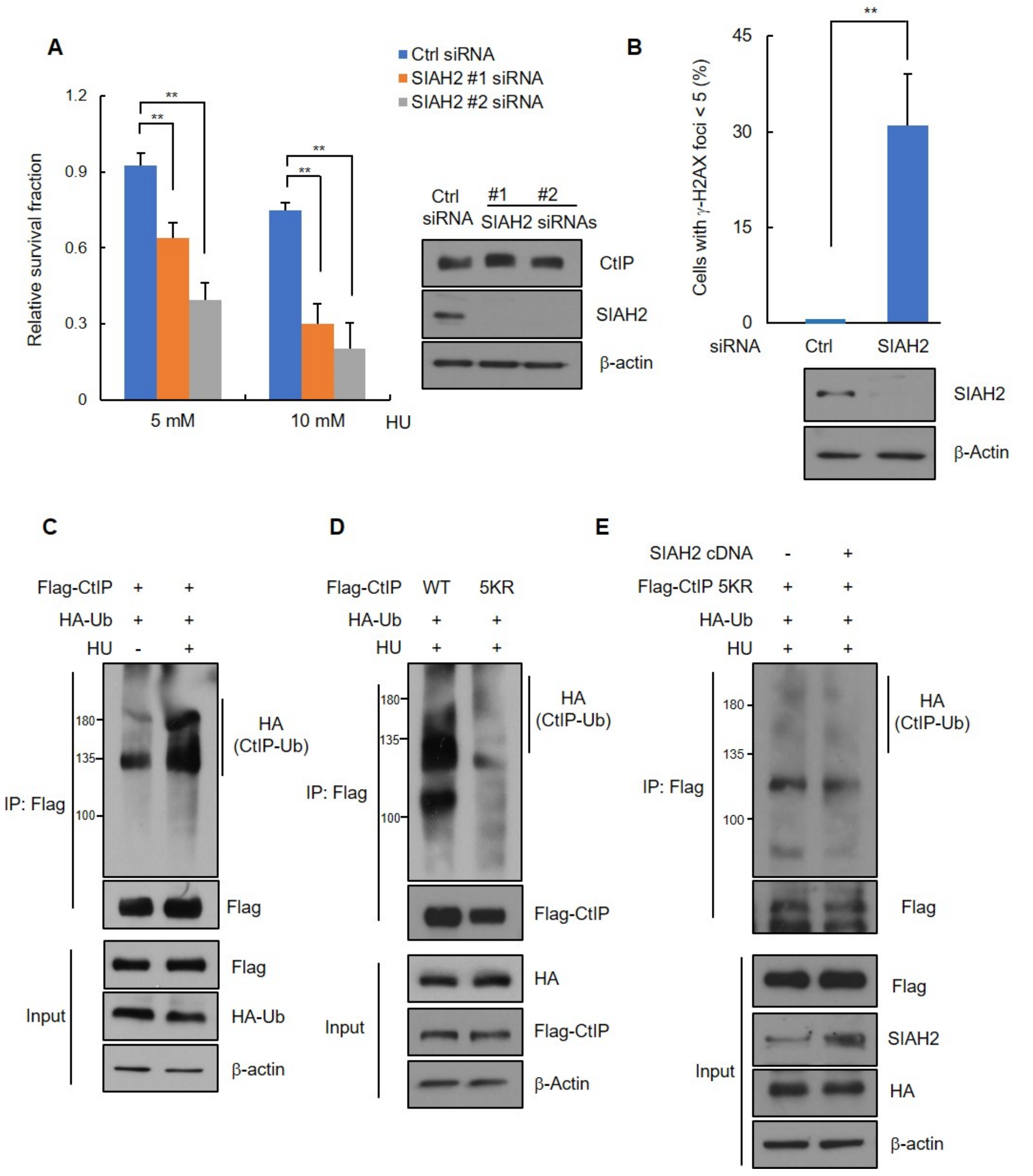

Figure S6. CtIP is ubiquitinated in response to replication stress.

(**A**) Colony-forming ability of control and SIAH2 knockdown cells treated with the indicated doses of HU for 3 h. Whole cell lysates were analyzed by immunoblotting using the indicated antibodies. Survival data is presented as mean ± SD (n = 3), ** $P < 0.01$. Statistical analysis was performed with the two-tailed Student's *t*-test. (**B**) Control- and SIAH2-depleted HeLa cells were treated with

3 mM HU for 24 h and then fixed and immunostained for γ-H2AX. The percentage of cells with > 5 foci for γ-H2AX is shown. Data represent mean ± SD (n = 3), ** $P < 0.01$. Statistical analysis was performed with the two-tailed Student's *t*-test. (**C**) HEK293T cells transfected with HA-ubiquitin and Flag-CtIP were treated with or without 3 mM HU. 3 h later, total cell lysates were immunoprecipitated with anti-Flag antibody. The immunoprecipitates were then blotted with the indicated antibodies. (**D**) HEK293T cells were cotransfected with HA-ubiquitin along with Flag-CtIP WT or Flag-CtIP-5KR, treated with 3 mM HU for 3 h, and subjected to immunoprecipitation and immunoblotting as indicated. (**E**) Flag-CtIP-5KR-expressing HEK293T cells were transfected with HA-ubiquitin with or without SIAH2 and treated with 3 mM HU. 3 h later, total cell lysates were immunoprecipitated with anti-Flag antibody and immunoblotted with the indicated antibodies.

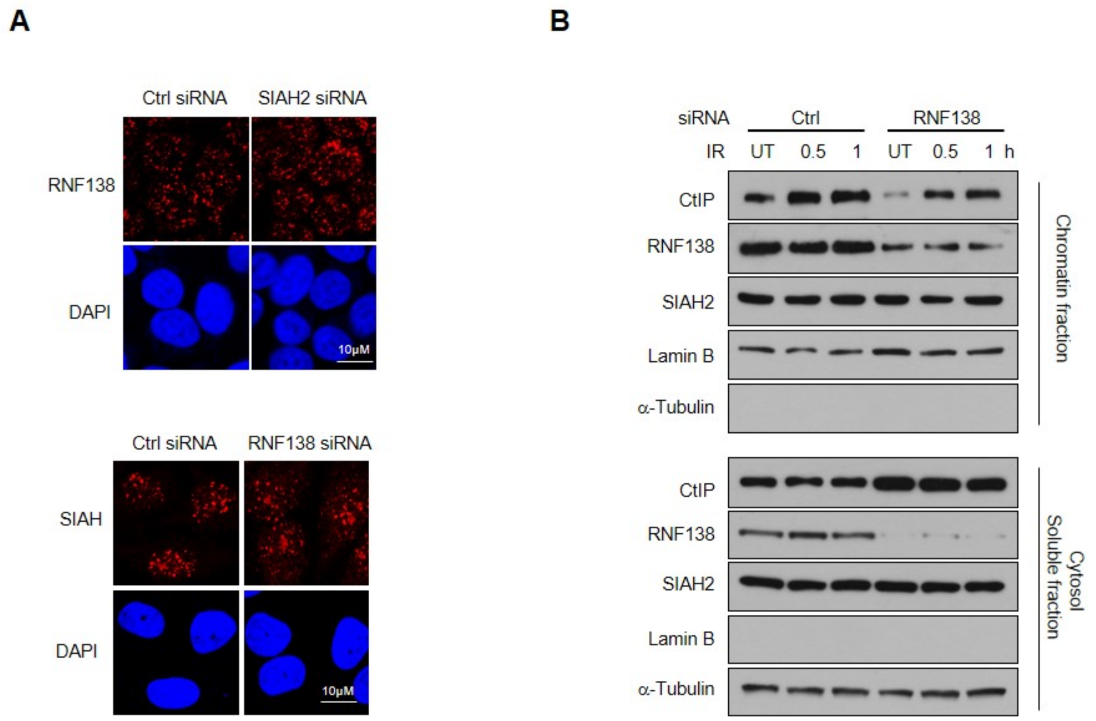

Figure S7. The effect of RNF138 knockdown on IR-induced SIAH2 foci formation and chromatin retention.

(**A**) HeLa cells cotransfected with HA-ubiquitin and either a scrambled negative control siRNA or siRNAs against SIAH2 (left) or RNA138 were treated with 5 Gy IR at 1 h, and immunostained with anti-RNF138 or anbti-SIAH2 antibodies. DNA was stained with DAPI. (**B**) Control and RNF138-depleted HeLa cells were either untreated or treated with 5 Gy IR. At the indicated time point after IR, chromatin and soluble fractions were extracted and analyzed by western blotting.

Table S1 List of primer sequences used for RT-PCR, ChIP and cloning.

Gene	Forward primer sequence	Reverse primer sequence	Used
HA CtIP WT	CGGATATCCGATGAACATCTTGGGA	ACGCGTCGACCTATGTCTTCTGCTC	cloning
HA CtIP-500	CGCGAATTCATTCAGCGTCAAGAG	CGCCTCGAGCTATGTCTTCTGCTCCTT	cloning
HA CtIP-600	CGCGAATTCTTAGATGACATAAAGAGTG C	CGCCTCGAGCTATGTCTTCTGCTCCTT	cloning
HA CtIP-700	CGCGAATTCCTCTTAAAAATGAAGAAGC	CGCCTCGAGCTATGTCTTCTGCTCCTT	cloning
HA CtIP-800	CGCGAATTCCGGAAAAAAGAGGAG	CGCCTCGAGCTATGTCTTCTGCTCCTT	cloning
Flag SIAH2 ΔRING	1. GCGATCGC ATGAGCCGCCCGT<br>2.GGCGCCCTGACGCCCAGCATCA	1.CGAAGAGCGAGGTCAGCTCGT<br>2.ACGCGTTGGACAACATGTAGA	cloning
Flag SIAH2 ΔZINC	1.GCGATCGC ATGAGCCGCCCGT<br>2.AGCATTACCACCCTTCAGGGA	1.CGAGGCCACCTTCTCCATAGC<br>2.ACGCGTTGGACAACATGTAGA	cloning
Flag SIAH2 ΔSBD	GCGATCGC ATGAGCCGCCCGTC	AACGCGTACGCGTTGGACAACA	cloning
GST SIAH2 SBD	AAAGAATTCCACGCCCACAAGAGCA	GCGGCCGCATGTAGAAATAGTAACA	cloning
GST SIAH2 ΔSBD	AAAGAATTCCACGCCCACAAGAGCA	AAAAGCGGCCGCTGGACAACA	cloning
GST SIAH2 WT	AAAGAATTCATGAGCCGCCCGTCCTCCA C	GCCCTCGAGTGGACAACATGTAGAAAT AG	cloning
GST CtIP 700-799	CGCGAATTCCTCTTAAAAATGAAGAAGC	ATTAAGCGGCCGCTTTCTTCTCTCC	cloning
GFP-SIAH1	AAACTCGAGATGAGCCGTCAGACT	CCCGAATTCCCACACATGGAAATA	cloning
No DSB	ATTGGGTATCTGCGTCTAGTGAGG	GACTCAATTACATCCCTGCAGCT	RT-PCR
DSB-335nt	GAATCGGATCTATGCGACTGATC-3	TTCCAAAGTTATTCCAACCCGAT	RT-PCR
DSB-1618nt	TGAGGTGACATTAGAACTCAGA	AGGACTCACTTACACGGCCTTT	RT-PCR
DSB-3500nt	TCCTAGCCAGATAATAATAGCTATACAA ACA	TGAATAGACAGACAACAGATAAATGA GACA	RT-PCR
ISec1 180	CATGCCCGAAGGCTACGT	CGGCGCGGGTCTTGTA	ChIP
Flag CtIP 5KR	GAAGAATTCTTCACCAGAAATCAACAGC TGAGGG (K62R)	CCCTCAGCTGTTGATTCTTGGTGAAGA ATTCTTC (K62R)	cloning
	GTCCTTCATGAAACCATTAGAGTTTTAG AAGATCGG (K78R)	CCGATCTTCTAAAACTCTAATGGTTTCA TGAAGGAC (K78R)	cloning
	CCGGCAGCAGAATCTTAGACTTATTACA GAACTT (K115R)	AAGTTCTGTAATAAGTCTAAGATTCTG CTGCCGG (K115R)	cloning
	CTACAGGAAGAAAATAGAAGGCTTTCTG AACAACTCC (K132/133R)	GGAGTTGTTCAGAAAGCCTTCTATTTTC TTCCTGTAG (K132/133R)	cloning
Flag-CtIP-6KR	TGCTCTCCAGACAATCGACCATCATTAC AAATA (K572R)	TATTTGTAATGATGGTCGATTGTCTGGA GAGCA (K572R)	cloning
	CCATCATTACAAATAAGAGAAGAAAATG CTGTC (K578R)	GACAGCATTTTCTTCTCTTATTTGTAAT GATGG (K578R)	cloning
	AGAACTGGTAAAATAAGGTCTCTACAAA ACAAC (K640R)	GTTGTTTTGTAGAGACCTTATTTTACCA GTTCT (K640R)	cloning
	GTCTACTGCCACAAGGAGACTACACACT CATG (K759/760R)	CATGAGTGTGTAGTCTCCTTGTGGCAG TAGAC (K759/760R)	cloning
	GTGGAGCCGTATTTTAGAGGTGATGAAA GAGAG (K782R)	CTCTCTTTCATCACCTCTAAAATACGGC TCCAC (K782R)	cloning

Table S2. List of antibodies used in this study: WB, IP, IF.

Antibody	Species	Application	Cat. No	Suppliers
SIAH2	rabbit, human	WB, IP, IF	PAB8469	Abnova
SIAH1	rabbit, human	WB	PAB10383	Abnova
CtIP	monkey, human	WB, IP	9201S	Cell signaling
CtIP	human	ChIP	61141	Active motif
CtIP	mouse, human	IF	sc-5970	Santa Cruz
CtIP	mouse, human	WB	sc-271339	Santa Cruz
CtIP	human	WB	a kind gift from professor Richard Baer, university of Texas Southwestern Medical Center	
Flag	mouse, human	WB, IP	F7425	Sigma-Aldrich
HA	rabbit, human	WB, IP	3724S	Cell Signaling
HA	rabbit, human	WB, IP	NB600-363	NOVUS Biologicals
GFP	rabbit, human	WB	NB600-308	NOVUS Biologicals
RAD51	mouse, human	IF	sc-8349	Santa cruz
RPA32-RPA2	mouse, human	IF	61184	Abcam
RPA	hamster, human	IF	NA18	Calbiochem
γ–H2A.X	Vertebrates	IF	05-636	Millipore
α-Tubulin	mouse, human	WB	LF-MA0117	Abfrontier
β-Actin	mouse, human	WB	sc-47778	Santa cruz
Cyclin A	mouse, human	IF	sc-271682	Santa cruz
Lamin B	mouse, human	WB	sc-6216	Santa cruz
RNF138	human	WB	92730	Abcam
BRCA1	mouse, human	WB	A301-377A	Bethyl
Peroxidase- Affinipure Goat Anti-mouse IgG Fc gamma fragment	mouse, human	WB	115-035-008	Jackson
Peroxidase- Affinipure Goat Anti-rabbit IgG Fc gamma fragment	mouse, human	WB	115-035-008	Jackson
Alexa Fluor® 488 chicken anti-rabbit IgG	mouse, human	IF	A-21441	Invitrogen
Alexa Fluor® 488 chicken anti-mouse IgG	mouse, human	IF	A-21200	Invitrogen
Alexa Fluor® 594 chicken anti-rabbit IgG	mouse, human	IF	A-21442	Invitrogen
Alexa Fluor® 594 chicken anti-mouse IgG	mouse, human	IF	A-21201	Invitrogen
Alexa Fluor® 594 chicken anti-goat IgG	mouse, human	IF	A-21468	Invitrogen
IgG from mouse serum	mouse, human	IP, IF	I5381	Sigma-Aldrich
IgG from Rabbit serum	mouse, human	IP, IF	I5006	Sigma-Aldrich
